# National trends in oncology specialists’ EHR inbox work, 2019-2022

**DOI:** 10.1093/jnci/djaf052

**Published:** 2025-03-03

**Authors:** A Jay Holmgren, Nate C Apathy, Jennie Crews, Tait Shanafelt

**Affiliations:** Division of Clinical Informatics and Digital Transformation, University of California San Francisco, San Francisco, CA, United States; School of Public Health, University of Maryland, College Park, MD, United States; Department of Medicine, Stanford University, Palo Alto, CA, United States; Department of Medicine, Stanford University, Palo Alto, CA, United States

## Abstract

**Background:**

Electronic health record (EHR) burden is an important driver of the ongoing physician burnout crisis. In particular, EHR-based messaging (also known as “inbox”)—including messages from patients—is associated with burnout and decreased well-being. Little is known about EHR messaging burden for oncologists. To address this gap, we assessed trends in oncologist EHR messaging volume and EHR time from 2019 to 2022 across oncology subspecialties.

**Methods:**

This study used EHR metadata for all US oncology physicians (including medical oncologist/hematologists, radiation oncologists, pediatric oncologists, gynecologic oncologists, and surgical oncologists) providing ambulatory care using an Epic EHR system to measure inbox volume and EHR time from July 2019 through April 2022. Descriptive statistics and multivariable regression were used to evaluate differences over time and across subspecialties.

**Results:**

This sample of 15 653 oncology physicians across 43 228 633 ambulatory visits found that message volume for oncologists increased 19.0% from 2019 to 2022, and patient-initiated messages increased 34.0%. The EHR time increased 16.2% from 2019 to 2022, whereas EHR “work outside of work” time increased 12.1%. Medical oncologist/hematologists had the highest inbox volume, patient-message volume, and EHR time of oncology subspecialists.

**Conclusion:**

Rising levels of EHR work and message volume among oncology physicians are concerning given the role of EHR burden in physician burnout. Oncologists have seen increased EHR time and message volume, especially patient-initiated messages, following the onset of the COVID-19 pandemic. Health system leaders and policymakers should invest in efforts to reduce EHR and inbox burden for all oncologists, with a focus on physicians with the greatest burden.

## Introduction

The onset of the COVID-19 pandemic caused dramatic changes to the structure of ambulatory care delivery, including a rapid increase in the number of asynchronous secure messages sent by patients to their physicians.^1^^,^^2^ The rapid increase in patient-initiated messages has strained physician capacity to manage electronic health record (EHR)–based messaging, known as “inbox,” while dealing with already-high levels of EHR work.^3^^,^^4^ This EHR burden, especially time spent outside of work hours, is associated with burnout,^5-7^ and excessive inbox messaging may be especially harmful to physician well-being.^8^^,^^9^ The result is an ongoing crisis that has been referred to as “death by patient portal.”^10^

Oncologists may be particularly susceptible to inbox burden because they manage complex patients with numerous care touchpoints, resulting in messages from external (patient) and internal (other clinicians, test result notifications) sources. However, to date, there is little national evidence for the state of oncologists EHR inbox volume and whether the broad increase in EHR inbox work and time spent in the EHR system has affected oncologists to the same degree as other specialties. Further, it is likely that inbox burden and EHR work is not evenly distributed across oncology subspecialties given the differences in their practice patterns and their patients care needs. For example, although there is substantial evidence that primary care physicians have greater EHR burden relative to an aggregate group of “medical subspecialists,”^3^^,^^4^ it may be that medical oncologists/hematologists, who often play a role similar to a primary care physician’s coordinating role for their patients while simultaneously treating those patients’ cancer, have a greater EHR burden relative to other subspecialties. Understanding how EHR message volume and the resulting time spent working in the her system has changed over time and across oncology subspecialties is critical to the design of health system interventions and policies to address EHR-related burnout and physician well-being. For example, if certain oncology subspecialties are subject to particularly high EHR inbox burden, resources such as staff support for inbox management should be allocated to the most burdened physicians first, and new technologies, such as artificial intelligence to draft replies to patient messages should be developed, tested, and refined with the most burdened physicians in mind.^11^

To address these gaps in knowledge, we set out to answer 3 research questions to better understand the dynamics of EHR inbox volume and EHR work for oncology physicians. First, how has EHR inbox volume, both overall and messages initiated by patients (which are likely to require clinical expertise and decision making), changed over time? Second, how has EHR time, both overall and the subset of time that takes place outside clinic hours, changed over time? Finally, how do these measures of EHR work vary across oncology subspecialties, and which oncology-related subspecialties face the highest EHR inbox and overall time burden? We used national EHR metadata to provide detailed insights into the state of oncologist EHR inbox work and overall EHR time over time to guide health system leaders, policymakers, and practicing physicians in effectively targeting their EHR inbox burden relief efforts.

## Methods

### Data

Deidentified EHR metadata were extracted from the Epic Signal platform, which aggregates measures of clicks, keystrokes, and mouse movement within the her system. All time-based measures include “active time” in the EHR system, which stops counting after 5 seconds of no mouse movement, keystrokes, or clicks.^12^ This time is tracked across each component of EHR work and broken out into time spent on EHR messaging in the inbox (InBasket); time spent on documentation; time spent on clinical (chart) review; time spent on orders; and other time, including time spent in patient navigator and other EHR functions. Signal also tracks discrete actions, including the number of messages each physician receives, broken down by message type.^9^ The sample included monthly deidentified multi-institution data from July 2019 through April 2022 for all oncology physicians using Epic for outpatient care in the United States, including data on physician subspecialty (defined using Epic’s Signal mapping, with full specialty crosswalk in Table S1), practice setting, and number of weekly visits. Physician assistants and nurse practitioners were excluded from this analysis specifically to evaluate physician workflows. All measures were normalized to weekly averages to account for varying month length. This study was deemed nonhuman subjects research for using fully deidentified data by the University of California San Francisco Institutional Review Board.

### Measures

#### EHR messaging volume

The first EHR-based outcome measure was volume of messages received by oncology physicians. To determine overall message volume, we summed all weekly inbox messages oncology physicians received. We then measured the subset of messages from patients, including (1) patient medical advice request through the portal and (2) phone calls that were forwarded to the physician EHR inbox. Combined, these message subtypes represent the totality of patient-initiated inbox messages that physicians received for clinical issues. Only messages received or addressed by the physician were included, whereas messages sent to shared pools and completed by another clinician were excluded.

#### EHR time

The second EHR-based outcome measure was time spent in the EHR system by oncology physicians. To assess EHR time, we measured total weekly EHR time, the subset of EHR time spent working in InBasket, and EHR “work outside of work” time. To construct our work outside of work measure, we combined EHR time on scheduled clinic days outside of scheduled patient care hours, with a 30-minute buffer before the first appointment and after the last appointment of the day, and EHR time on days without appointments. Measures included time dedicated to outpatient care only and excluded any time spent on inpatient as well as time on activities unrelated to patient care (eg, time spent in Epic modules such as SlicerDicer for research or operational reporting). We also reported each component of work outside of work (time on unscheduled days and time outside of scheduled hours on scheduled days) separately in our regression models as a robustness test.

### Analysis

#### Descriptive statistics

First, we used descriptive statistics to calculate message volume and total EHR time over time by subspecialty across 5 oncology subspecialties—medical oncology/hematology, pediatric oncology, radiation oncology, gynecologic oncology, and surgical oncology—visualized as line graphs to show changes over time by oncology subspecialty.

#### Multivariable regression models

To compare our EHR-based outcomes over time as well as oncology subspecialties to each other while adjusting for potential confounders, including workload and practice setting, we created 5 multivariable ordinary least square regression models, 1 for each message and EHR time outcome variable. Our independent variables of interest were binary indicator variables for each year in our sample to assess changes over time for all oncology physicians relative to 2019 as well as binary indicator variables for each oncology subspecialty, with controls for practice setting (academic vs nonacademic) and number of outpatient visits and additional controls for calendar month fixed effects to control for seasonality. For oncology subspecialties, radiation oncology was used as the reference group because these physicians had the lowest EHR inbox workload. Each coefficient can therefore be interpreted as the mean difference between radiation oncologists and oncologists in that subspecialty. We also conducted robustness tests using EHR outcomes per encounter in all multivariable models. All models used robust SEs clustered at the physician level.

## Results

### Sample characteristics

The final analytic sample included 15 653 unique physicians over a 33-month study period, covering 43 228 633 visits and 380 879 physician-month observations. This sample included 9600 medical oncology/hematology physicians (61.3% of the sample), 3138 (20.0%) radiation oncologist physicians, 1166 (7.5%) pediatric oncology/hematology physicians, 907 (5.8%) gynecologic oncologist physicians, and 842 (5.4%) surgical oncologist physicians. In total, 12 113 (77.1%) physicians practiced in academic settings, with the remainder in nonacademic settings; 3605 (22.9%) practiced in the Northeast US Census region, 4210 (26.8%) in the South, 4221 (26.9%) in the Midwest, and 3681 (23.4%) in the West (Table 1).

**Table 1.** Sample descriptive characteristics.

Characteristic	No. (%)
Unique physicians	15 653 (100)
Physicians, by subspecialty	
Radiation oncology	3138 (20.0)
Medical oncology/hematology	9600 (61.1)
Pediatric oncology	1166 (7.4)
Gynecologic oncology	907 (5.8)
Surgical oncology	842 (5.4)
Physician-month observations	382 447 (100)
Practice setting	
Academic practice setting	12 113 (77.1)
Nonacademic setting	3604 (22.9)
Region	
Midwest	4221 (26.9)
Northeast	3605 (22.9)
South	4210 (26.8)
West	3681 (23.4)

### Message volume

**Figure 1.**
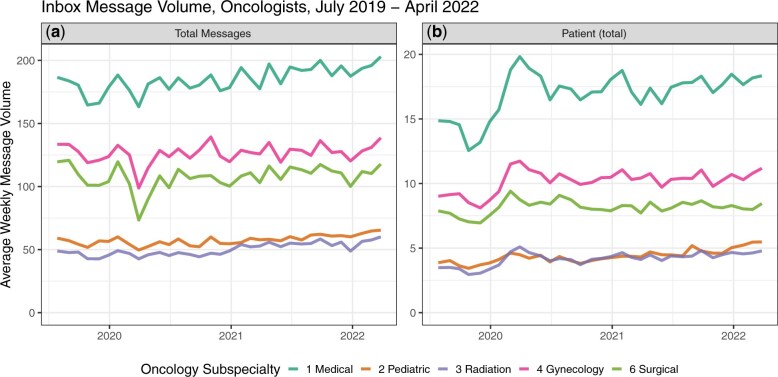
Inbox message volume, oncologists, July 2019 to April 2022. Panel A represents total message volume while Panel B represents the sub-set of message volume that includes patient medical advice request messages and patient phone calls.

Mean overall EHR inbox messages received by physicians across all oncology specialties in 2019 was 127.4 per week compared with 151.6 in 2022, a 19.0% increase. Medical oncology/hematology physicians had the highest message volume, with 186.5 weekly messages in July 2019, increasing to 203.0 in April 2022 (Figure 1, A). Gynecologic oncology physicians (133.5 mean weekly messages in July 2019 rising to 138.6 in April 2022) received the second-most messages, followed by surgical oncologists (119.6 mean weekly messages rising to 117.9 in April 2022), pediatric oncologists (59.1 in July 2019 rising to 65.4 in April 2022), and radiation oncologists (48.9 mean weekly messages in July 2019 rising to 60.1 in April 2022).

The mean number of overall patient-initiated messages oncology physicians received increased from 10.0 weekly in 2019 to 13.4 weekly in 2022, a 34.0% increase. Across oncology subspecialties, medical oncologist/hematologist physician message volume increased 22.8%, from 14.9 mean weekly messages in July 2019 to 18.3 in April 2022. The number of messages gynecologic oncologists received rose 24.4%, from 9.0 to 11.2 patient-initiated messages per week, whereas the number of messages surgical oncologists received rose from 7.9 to 8.4 (a 6.3% increase). The number that pediatric oncology physicians received rose from 3.9 to 5.5 (a 41.0% increase), and the number that radiation oncologists received increased 37.1%, from 3.5 to 4.8 patient-initiated messages per week (Figure 1, B).

### EHR time

For all oncology disciplines, total EHR time increased from a mean of 400.2 minutes per week in 2019 to 465.2 in 2022 (a 16.2% increase). InBasket time rose from 47.2 to 59.2 minutes per week (a 25.4% increase), whereas EHR work outside of work rose from a mean of 189.0 minutes per week in 2019 to 211.8 in 2022 (a 12.1% increase).

**Figure 2.**
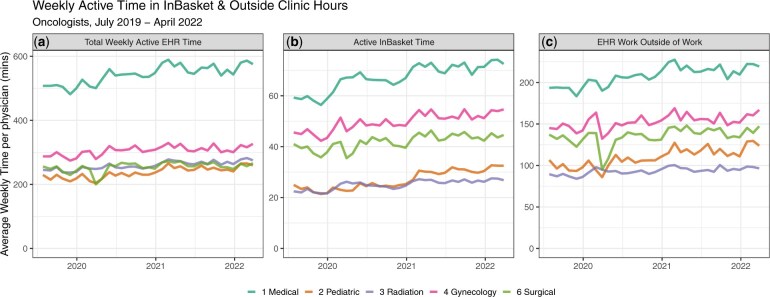
Weekly activity in InBasket and outside clinic hours. Panel A shows total weekly active EHR time. Panel B shows active inbasket time. Panel C shows EHR work outside of work time.

Medical oncologists/hematologists had the highest total weekly EHR time (July 2019 = 507.6 minutes per week; April 2022 = 575.5 minutes per week—a 13.4% increase) (Figure 2, A); followed by gynecologic oncology (July 2019 = 287.4 minutes; April 2022 = 326.4 minutes—a 13.6% increase); and then surgical, radiation, and pediatric oncology. Medical oncologists/hematologists had the highest InBasket time, rising from 59.3 minutes per week in July 2019 to 72.5 minutes per week in April 2022 (Figure 2, B) as well as the highest EHR work outside of work at a mean of 193.6 minutes per week in July 2019 rising to 219.4 minutes per week in April 2022 (a 13.3% increase) (Figure 2, C). Full numerical results are available in Table S2.

### Multivariable regression results

The descriptive univariate differences in total message volume and EHR time, both over time and across oncology subspecialties, persisted even after adjusting for outpatient visit volume and practice setting. Total message volume increased by 15.9 messages per week from 2019 to 2022, whereas patient-initiated messages increased by 2.7 per week. Total EHR time increased by 49.5 minutes per week, whereas EHR InBasket time rose by 10.9 minutes and EHR work outside of work time increased by 20.4 minutes by 2022 compared with 2019 (all *P*s < .001) (Table 2).

**Table 2.** Factors associated with oncologist inbox and EHR burden.^a^

Factor Sub-Specialty	Total weekly messages, coefficient^b^ (95% CI)	Weekly patient messages, coefficient^b^ (95% CI)	Total EHR min/wk, coefficient^b^ (95% CI)	InBasket min/wk, coefficient^b^ (95% CI)	EHR work outside of work min/wk, coefficient^b^ (95% CI)
Radiation oncology	(Referent)	(Referent)	(Referent)	(Referent)	(Referent)
Medical oncology/hematology	121.4^c^ (117.8 to 124.9)	12.1^c^ (11.6 to 12.6)	255.2^c^ (245.6 to 264.7)	39.7^c^ (38.1 to 41.3)	107.7^c^ (102.3 to 113.1)
Pediatric oncology	66.3^c^ (60.8 to 71.9)	4.1^c^ (3.4 to 4.8)	112.6^c^ (96.7 to 128.5)	15.5^c^ (13.1 to 17.9)	44.9^c^ (36.7 to 53.1)
Gynecologic oncology	95.9^c^ (90.8 to 101.0)	7.3^c^ (6.3 to 8.3)	91.2^c^ (77.4 to 105.0)	29.2^c^ (26.5 to 32.0)	69.2^c^ (60.5 to 77.9)
Surgical oncology	86.6^c^ (81.4 to 91.8)	6.0^c^ (5.0 to 6.9)	62.1^c^ (49.1 to 75.1)	23.9^c^(21.2 to 26.6)	55.5^c^ (47.4 to 63.6)
**Academic status**					
Nonacademic organization	(Referent)	(Referent)	(Referent)	(Referent)	(Referent)
Academic organization	‒24.0^c^ (‒28.6 to ‒19.3)	‒3.7^c^ (‒4.7 to ‒2.8)	‒74.2^c^ (‒85.9 to ‒62.5)	‒13.3^c^ (‒15.8 to ‒10.7)	4.8 (‒1.9 to 11.6)
**No. of visits**					
Total weekly visits (marginal effect of each additional visit)	3.4^c^ (3.1 to 3.7)	0.2^c^ (0.2 to 0.2)	7.6^c^ (6.9 to 8.3)	0.7^c^ (0.7 to 0.8)	1.7^c^ (1.4 to 1.9)
**Year**					
2019	(Referent)	(Referent)	(Referent)	(Referent)	(Referent)
2020	5.8^c^ (4.6 to 6.9)	2.8^c^ (2.6 to 3.0)	33.4^c^ (31.1 to 35.6)	6.5^c^ (6.1 to 6.9)	12.1^c^ (10.6 to 13.6)
2021	11.1^c^ (9.5 to 12.7)	2.6^c^ (2.3 to 2.8)	44.9^c^ (41.5 to 48.2)	9.8^c^ (9.2 to 10.4)	19.2^c^ (17.0 to 21.3)
2022	15.9^c^ (13.6 to 18.2)	2.7^c^ (2.4 to 3.0)	49.5^c^ (44.5 to 54.5)	10.9^c^ (10.0 to 11.8)	20.4^c^ (17.2 to 23.6)

Abbreviations: CI = confidence interval; EHR = electronic health record.

a 380 878 observations for all models.

b Coefficients can be interpreted as the difference in either weekly messages or minutes per week of EHR time compared with the reference category (eg, radiation oncology). All dependent variables are measured weekly. All models included calendar month fixed effects not shown to control for seasonality. Models include robust SEs clustered at the physician level.

c *P* < .001.

Across our EHR use measures, medical oncology/hematology physicians had the highest total EHR inbox message volume (121.4 messages per week greater than the reference group, radiation oncologists), patient-initiated message volume (β = 12.1 messages per week), total EHR time (β = 255.2 minutes per week), InBasket time (β = 39.7 minutes per week), and EHR work outside of work time (β = 107.7 minutes per week). Gynecologic oncology physicians had the next-highest overall inbox volume (β = 95.9), patient-initiated message volume (β = 7.3), total EHR time (β = 91.2), InBasket time (β = 29.2), and work outside of work time (β = 69.2) (all *P*s < .001). Across all measures, we found similar results when specifying the dependent variable per encounter (Table S3) and when disaggregating work outside of work into time outside of scheduled hours on days with scheduled visits (ie, EHR work after work on a clinic day) and time on unscheduled days (ie, EHR work on nonclinic days and vacations/weekends) (Table S4).

## Discussion

In this national longitudinal study, we found large increases in oncologist EHR message volume, both total messages and messages originating from patients, as well as total EHR time, InBasket time, and EHR work outside of work between 2019 and 2022. Among oncology disciplines, medical oncology/hematology physicians received the most messages and spent nearly twice as much total time in the EHR system as other oncology subspecialties.

Our results have important implications for organizations working to address EHR burden and its contribution to high rates of occupational distress in oncology physicians. First, the post–COVID-19 increase in message volume and EHR time has durably increased burden through 2022 and represents the “new normal,” and these results suggest that it is unlikely that patients’ desire to communicate with their physicians through this channel and the associated work will decrease. Although the evidence for the relationship between patient outcomes and portal messaging is nascent, it should also be noted that high value and timely clinical care can be provided through such interactions,^13-15^ with some evidence that physicians who spend greater time interacting with their patients through the EHR have better patient outcomes.^16^ Health systems should recognize these facts and develop sustainable workflows and approaches to appropriately recognize and value EHR-related work. This shift may require the allocation of resources to enable new models of team-based inbox management, with associated staffing, to help prevent physicians becoming overwhelmed by this increasingly popular modality of care. Technology solutions such as artificial intelligence may also alleviate physician inbox burden, though early evaluations of generative artificial intelligence deployed to draft message replies have not shown substantial reductions in physician EHR time dedicated to inbox work^11^ and are unlikely to fully replace high-performing care teams in the near future. Second, previous studies suggesting that primary care physicians have higher EHR burden than other physicians when specialty physicians are pooled have not fully accounted for variation within specialties. Notably, medical oncology/hematology physicians receive more messages and spend twice as much time in the EHR system compared with previous estimates that aggregated a broad set of medical specialists.^3^ Contrary to previous estimates that primary care physicians receive “5 times more patient-initiated messages” than other specialists, we find that medical oncology/hematology physicians received more than 18 patient-initiated messages per week compared with 34 for primary care physicians during the same period. Given the role EHR burden plays in the physician burnout crisis, the association between burnout and decreased clinical productivity and turnover,^17-19^ and the projected oncology workforce shortage,^20^ health systems and care delivery organizations must address the EHR burden of oncologists. Interventions such as support staff for message triage and response,^21^ policies to manage patient expectations for messaging,^10^ and protected time for inbox work are necessary to create sustainable workloads. Investment in these interventions have great promise to reduce burnout and pay dividends by reducing the number of oncologists leaving or reducing practice^22^ and improving productivity.^17^^,^^23^

Our results also have important implications for policymakers and the nature of technology-based change in health-care policy. One key reason why patient-initiated messages are particularly burdensome is that they represent a new modality of delivering care to patients that is not appropriately recognized or compensated in a fee-for-service environment, underscoring the critical friction between existing payment policy and structure and the changing nature of care delivery, including the growing role of EHR-based work. Billing codes developed for paper-based workflows and phone calls likely underappreciate the documentation, chart review, patient messaging tasks, and between-visit care delivered asynchronously in the EHR era. The resulting EHR work outside of work those tasks generate is considerable—more than 3.5 hours of active EHR use per week for oncologists in 2022—and may be particularly burdensome for specialists managing complex, high-need patients. In this regard, the approaches used to address the EHR inbox burden for oncology specialists may differ from those used in primary care, where new proposals for capitated or other population-based payment models may encourage practices to provide high-value care through nontraditional care models. Although billing codes established in 2020 are broadly in use by payers, including Medicare,^24^ they have not seen much use by practicing physicians.^25^ This is likely due to a confluence of factors, such as physician concerns over harming the patient–physician relationship or exacerbating the financial toxicity of high-cost care,^26^ but the existing implementation of fee-for-service billing codes is cumbersome and requires substantial additional work (eg, assessing whether the messages meet billing criteria, going through the steps to associate a diagnosis and attach a billing code to a message, and documenting that charge) and have many restrictions (ie, physicians cannot bill for a message 3 days before and 7 days after an in-person visit). Due in part to these administrative frictions, organizations that have empowered physicians to bill for inbox messages have reported that less than 1% of inbox message threads are billed.^27^ Even if inbox billing use were to increase, the current reimbursement for care delivered in this way is woefully inadequate to support the care team structure necessary to do so.^27^ Without a sustainable reimbursement system, health systems are disincentivized from investing in supporting physician inbox work and instead may push patients toward higher-cost, less timely care, such as scheduled visits. Policymakers and regulators should explore alternative methods for reimbursing physicians for asynchronous care delivered through the EHR patient portal, such as partial capitation or even automated reporting of patient-initiated inbox volume, to ensure that physicians can develop sustainable workflows that incorporate asynchronous, secure messaging while ensuring patient access to care.

### Limitations

Our study results should be interpreted with some limitations in mind. First, we use data from a single EHR vendor, though Epic has the largest market share in the United States and is used by a wide variety of care delivery organizations.^28^ Second, our data include only outpatient EHR time. Although the majority of concerns regarding inbox burden center around ambulatory care, we were unable to assess changes in EHR use in the inpatient setting or determine physicians’ allocation of time between inpatient and ambulatory care. Third, although our data enable us to granularly measure time spent in the EHR system outside of scheduled hours or on unscheduled days, we were unable to identify other, specific dimensions of EHR work that may contribute to burnout, such as EHR time spent on weekends or vacation. Fourth, although we include all physicians classified as oncologists in Epic’s Signal EHR metadata platform and our study aimed to directly compare EHR and inbox workload for physicians who primarily treat patients with cancer, our methodology is likely to exclude some physicians who may treat patients with patients as part of their work, such as otorhinolaryngologists or other surgeons, and our subcategory of “surgical oncologists” is a relatively small group of physicians. Finally, our EHR metadata do not contain direct measures of burnout. However, several studies have found EHR time, work outside of work, and especially InBasket time and inbox volume to be associated with burnout.^7-9^

US oncology physicians saw a large increase in message volume and EHR time between 2019 and 2022. Medical oncology/hematology physicians had the highest message and EHR time burden among oncology subspecialties. Efforts to address excessive EHR inbox work should target the most burdened physicians, including medical oncology/hematology physicians. Meaningful progress will require action by health-care organizations and payors, and policymakers interested in ensuring both the stability of the physician workforce and patient access to care should consider exploring alternative payment models that support the efficient use of asynchronous written care.

## Supplementary Material

djaf052_Supplementary_Data

## Data Availability

Data is proprietary to the data provider, Epic Systems. Data are available upon request from the authorship team with permission from Epic.

## References

[1] Nath B , Williams B, Jeffery MM, et al Trends in electronic health record inbox messaging during the COVID-19 pandemic in an ambulatory practice network in New England. JAMA Netw Open. 2021;4:e2131490. 10.1001/jamanetworkopen.2021.31490 34636917 PMC8511977

[2] Holmgren AJ , Downing NL, Tang M, Sharp C, Longhurst C, Huckman RS. Assessing the impact of the COVID-19 pandemic on clinician ambulatory electronic health record use. J Am Med Inform Assoc. 2022;29:453-460. 10.1093/jamia/ocab268 34888680 PMC8689796

[3] Rotenstein LS , Holmgren AJ, Downing NL, Bates DW. Differences in total and after-hours electronic health record time across ambulatory specialties. JAMA Interl Med. 2021;181:863-865. 10.1001/jamainternmed.2021.0256 PMC7985815 33749732

[4] Overhage JM , McCallie D. Physician time spent using the electronic health record during outpatient encounters: a descriptive study. Ann Inter Med. 2020;172:169-174. Published online, 10.7326/M18-3684 31931523

[5] Gardner RL , Cooper E, Haskell J, et al Physician stress and burnout: the impact of health information technology. J Am Med Inform Assoc. 2019;26:106-114. 10.1093/jamia/ocy145 30517663 PMC7647171

[6] Hilliard RW , Haskell J, Gardner RL. Are specific elements of electronic health record use associated with clinician burnout more than others? J Am Med Inform Assoc. 2020;27:1401-1410. 10.1093/jamia/ocaa092 32719859 PMC7647296

[7] Dyrbye LN , Gordon J, O'Horo J, et al Relationships between EHR-based audit log data and physician burnout and clinical practice process measures. Mayo Clinic Proc. 2023;98:398-409. 10.1016/j.mayocp.2022.10.027 36868747

[8] Adler-Milstein J , Zhao W, Willard-Grace R, Knox M, Grumbach K. Electronic health records and burnout: Time spent on the electronic health record after hours and message volume associated with exhaustion but not with cynicism among primary care clinicians. J Am Med Inform Assoc. 2020;27:531-538. 10.1093/jamia/ocz220 32016375 PMC7647261

[9] Tai-Seale M , Dillon EC, Yang Y, et al Physicians’ well-being linked to in-basket messages generated by algorithms in electronic health records. Health Affairs. 2019;38:1073-1078. 10.1377/hlthaff.2018.05509 31260371

[10] Stillman M. Death by patient portal. JAMA. 2023;330:223-224. 10.1001/jama.2023.11629 37389857

[11] Garcia P , Ma SP, Shah S, et al Artificial intelligence–generated draft replies to patient inbox messages. JAMA Network Open. 2024;7:e243201. 10.1001/jamanetworkopen.2024.3201 38506805 PMC10955355

[12] Baxter SL , Apathy NC, Cross DA, Sinsky C, Hribar MR. Measures of electronic health record use in outpatient settings across vendors. J Am Med Inform Assoc. 2021;28:955-959. 10.1093/jamia/ocaa266 33211862 PMC8068413

[13] Reed ME , Huang J, Brand RJ, et al Patients with complex chronic conditions: Health care use and clinical events associated with access to a patient portal. PLoS One. 2019;14:e0217636. 10.1371/journal.pone.0217636 31216295 PMC6583978

[14] Alexander J , Beatty A. Association of patient portal messaging with survival among radiation oncology patients. Int J Radiat Oncol Biol Phys. 2024;120:627-638. 10.1016/j.ijrobp.2024.05.003 38723754

[15] Aljabri D , Dumitrascu A, Burton MC, et al Patient portal adoption and use by hospitalized cancer patients: a retrospective study of its impact on adverse events, utilization, and patient satisfaction. BMC Med Inform Decis Making. 2018;18:70. 10.1186/s12911-018-0644-4 PMC6062873 30053809

[16] Rotenstein LS , Holmgren AJ, Healey MJ, et al Association between electronic health record time and quality of care metrics in primary care. JAMA Netw Open. 2022;5:e2237086. 10.1001/jamanetworkopen.2022.37086 36255725 PMC9579903

[17] Shanafelt TD , Mungo M, Schmitgen J, et al Longitudinal study evaluating the association between physician burnout and changes in professional work effort. Mayo Clin Proc. 2016;91:422-431. 10.1016/j.mayocp.2016.02.001 27046522

[18] Hamidi MS , Bohman B, Sandborg C, et al Estimating institutional physician turnover attributable to self-reported burnout and associated financial burden: a case study. BMC Health Serv Res. 2018;18:851. 10.1186/s12913-018-3663-z 30477483 PMC6258170

[19] Shanafelt TD , Dyrbye LN, West CP, et al Career plans of US Physicians after the first 2 years of the covid-19 pandemic. Mayo Clin Proc. 2023;98:1629-1640. 10.1016/j.mayocp.2023.07.006 37923521

[20] Yang W , Williams JH, Hogan PF, et al Projected supply of and demand for oncologists and radiation oncologists through 2025: an aging, better-insured population will result in shortage. J Oncol Pract. 2014;10:39-45. 10.1200/JOP.2013.001319 24443733

[21] Sinsky CA , Shanafelt TD, Ripp JA. The Electronic Health Record Inbox: recommendations for Relief. J Gen Intern Med. 2022;37:4002-4003. 10.1007/s11606-022-07766-0 36036837 PMC9640509

[22] Shanafelt TD , Raymond M, Kosty M, et al Satisfaction with work-life balance and the career and retirement plans of US oncologists. J Clin Oncol. 2014;32:1127-1135. 10.1200/JCO.2013.53.4560 24616305 PMC4876331

[23] Shanafelt T , Goh J, Sinsky C. The business case for investing in physician well-being. JAMA Intern Med. 2017;177:1826-1832. 10.1001/jamainternmed.2017.4340 28973070

[24] Liu T , Zhu Z, Holmgren AJ, Ellimoottil C. National trends in billing patient portal messages as e-visit services in traditional Medicare. Health Affairs Scholar. 2024;2:qxae040. 10.1093/haschl/qxae040 38756169 PMC11034524

[25] Holmgren AJ , Oakes AH, Miller A, Adler-Milstein J, Mehrotra A. National trends in billing secure messages as e-visits. JAMA. 2024;331:526-529. 10.1001/jama.2023.26584 38198195 PMC10782378

[26] Judson TJ , Subash M, Harrison JD, et al Patient perceptions of e-visits: qualitative study of older adults to inform health system implementation. JMIR Aging. 2023; 6: E 45641. 10.2196/45641 PMC10257108 37234031

[27] Holmgren AJ , Byron ME, Grouse CK, Adler-Milstein J. Association between billing patient portal messages as e-visits and patient messaging volume. JAMA. 2023;329:339-342. 10.1001/jama.2022.24710 36607621 PMC10408262

[28] Holmgren AJ , Apathy NC. Trends in US hospital electronic health record vendor market concentration, 2012–2021. J Gen Intern Med. 2023;38:1765-1767. 10.1007/s11606-022-07917-3 36348217 PMC10212829

