# Supplementary material for: National trends in oncology specialists’ EHR inbox work, 2019-2022
Source: J Natl Cancer Inst. 2025 Mar 3;117(6):1253–9. doi: 10.1093/jnci/djaf052 (PMC12145914; doi:10.1093/jnci/djaf052)
Supplement: djaf052_Supplementary_Data [file djaf052_supplementary_data.pdf]

Supplementary Material

National Trends in Oncology Specialists' EHR Inbox Work, 2019 – 2022

Supplementary Table 11. Epic Signal EHR Metadata Standardized Specialty Mapping Crosswalk

Health System Reported Clinician Specialty	Mapped Sub-specialty used by Signal	Mapped Specialty used by Signal	Mapped Specialty Group used by Signal
Anesthesiology	Anesthesiology	Anesthesiology	Anesthesiology
Pediatric Anesthesiology	Pediatric Anesthesiology	Anesthesiology	Anesthesiology
Dental Oncology	Dentistry	Dentistry	Dentistry
Dental Radiology	Dentistry	Dentistry	Dentistry
Dentistry	Dentistry	Dentistry	Dentistry
Oral Medicine	Dentistry	Dentistry	Dentistry
Oral Pathology	Dentistry	Dentistry	Dentistry
Endodontics	Endodontics	Dentistry	Dentistry
Oral And Maxillofacial Surgery	Oral and Maxillofacial Surgery	Dentistry	Dentistry
Oral Surgery	Oral and Maxillofacial Surgery	Dentistry	Dentistry
Orthodontics	Orthodontics	Dentistry	Dentistry
Pediatric Dentistry	Pediatric Dentistry	Dentistry	Dentistry
Periodontics	Periodontics	Dentistry	Dentistry
Prosthodontics	Prosthodontics	Dentistry	Dentistry
Dermatology	Dermatology	Dermatology	Dermatology
Melanoma	Dermatology	Dermatology	Dermatology
Pediatric Dermatology	Pediatric Dermatology	Dermatology	Dermatology
Lab	Lab	Lab	Diagnostics
Lab Medicine	Lab	Lab	Diagnostics
Vascular Lab	Lab	Lab	Diagnostics
Cytopathology	Cytopathology	Pathology	Diagnostics
Dermatopathology	Dermatopathology	Pathology	Diagnostics
Pathology	Pathology	Pathology	Diagnostics
Surgical Pathology	Pathology	Pathology	Diagnostics
Pharmacology	Pharmacology	Pharmacology	Diagnostics
Pharmacy	Pharmacology	Pharmacology	Diagnostics
Interventional Radiology	Interventional Radiology	Radiology	Diagnostics
Neuro Interventional Radiology	Interventional Radiology	Radiology	Diagnostics
Vascular And Interventional Radiology	Interventional Radiology	Radiology	Diagnostics
Nuclear Medicine	Nuclear Medicine	Radiology	Diagnostics
Pediatric Radiology	Pediatric Radiology	Radiology	Diagnostics
Bone Density	Radiology	Radiology	Diagnostics

Dental Radiology	Radiology	Radiology	Diagnostics
Diagnostic Radiology	Radiology	Radiology	Diagnostics
Neuroradiology	Radiology	Radiology	Diagnostics
Radiology	Radiology	Radiology	Diagnostics
Radiology Ct	Radiology	Radiology	Diagnostics
Radiology Mr	Radiology	Radiology	Diagnostics
Medical Toxicology	Toxicology	Toxicology	Diagnostics
Toxicology	Toxicology	Toxicology	Diagnostics
Emergency Care	Emergency Medicine	Emergency Medicine	Emergency Care
Emergency Medicine	Emergency Medicine	Emergency Medicine	Emergency Care
Pediatric Emergency Medicine	Pediatric Emergency Medicine	Emergency Medicine	Emergency Care
Pediatric Urgent Care	Pediatric Urgent Care	Urgent Care	Emergency Care
Urgent Care	Urgent Care	Urgent Care	Emergency Care
Hospital Medicine	Hospital Medicine	Hospital Medicine	Hospital Medicine
Inpatient Nursing	Hospital Medicine	Hospital Medicine	Hospital Medicine
Pediatric Hospitalist	Hospital Medicine	Hospital Medicine	Hospital Medicine
Critical Care Medicine	Critical Care Medicine	Intensive Care Medicine	Hospital Medicine
Surgical Critical Care	Critical Care Medicine	Intensive Care Medicine	Hospital Medicine
Intensive Care	Intensive Care Medicine	Intensive Care Medicine	Hospital Medicine
Intensive Care Medicine	Intensive Care Medicine	Intensive Care Medicine	Hospital Medicine
Pediatric Critical Care Medicine	Pediatric Critical Care Medicine	Intensive Care Medicine	Hospital Medicine
Allergy	Allergy	Allergy and Immunology	Medical Specialties
Allergy And Immunology	Allergy and Immunology	Allergy and Immunology	Medical Specialties
Hiv	HIV	Allergy and Immunology	Medical Specialties
Hiv Program	HIV	Allergy and Immunology	Medical Specialties
Immunization Clinic	Immunology	Allergy and Immunology	Medical Specialties
Immunology	Immunology	Allergy and Immunology	Medical Specialties
Pediatric Allergy	Pediatric Allergy	Allergy and Immunology	Medical Specialties
Pediatric Immunology	Pediatric Immunology	Allergy and Immunology	Medical Specialties
Cardiac Rehabilitation	Cardiac Rehabilitation	Cardiology	Medical Specialties
Adult Congenital Heart Disease	Cardiology	Cardiology	Medical Specialties
Cardiology	Cardiology	Cardiology	Medical Specialties
Heart Transplant	Cardiology	Cardiology	Medical Specialties
Cardiovascular Disease	Cardiovascular Disease	Cardiology	Medical Specialties
Vascular Lab	Cardiovascular Disease	Cardiology	Medical Specialties
Advanced Heart Failure Transplant Cardiology	Heart Failure	Cardiology	Medical Specialties

Heart Failure	Heart Failure	Cardiology	Medical Specialties
Pediatric Cardiology	Pediatric Cardiology	Cardiology	Medical Specialties
Diabetes	Diabetes	Endocrinology	Medical Specialties
Diabetes Services	Diabetes	Endocrinology	Medical Specialties
Pediatric Diabetes	Diabetes	Endocrinology	Medical Specialties
Endocrinology	Endocrinology	Endocrinology	Medical Specialties
Endocrinology And Metabolism	Endocrinology	Endocrinology	Medical Specialties
Metabolism	Endocrinology	Endocrinology	Medical Specialties
Pediatric Diabetes	Pediatric Endocrinology	Endocrinology	Medical Specialties
Pediatric Endocrinology	Pediatric Endocrinology	Endocrinology	Medical Specialties
Endoscopy	Gastroenterology	Gastroenterology and Hepatology	Medical Specialties
Gastroenterology	Gastroenterology	Gastroenterology and Hepatology	Medical Specialties
Gastrointestinal Oncology	Gastroenterology	Gastroenterology and Hepatology	Medical Specialties
Gastroenterology And Hepatology	Gastroenterology and Hepatology	Gastroenterology and Hepatology	Medical Specialties
Hepatology	Hepatology	Gastroenterology and Hepatology	Medical Specialties
Pediatric Gastroenterology	Pediatric Gastroenterology	Gastroenterology and Hepatology	Medical Specialties
Genetics	Genetics	Genetics	Medical Specialties
Genetics Cancer Center	Genetics	Genetics	Medical Specialties
Medical Genetics	Medical Genetics	Genetics	Medical Specialties
Ped Genetics	Pediatric Genetics	Genetics	Medical Specialties
Pediatric Genetics	Pediatric Genetics	Genetics	Medical Specialties
Anticoagulation	Anticoagulation	Hematology	Medical Specialties
Blood Bank	Blood Bank	Hematology	Medical Specialties
Blood Banking Transfusion Medicine	Blood Bank	Hematology	Medical Specialties
Bone Marrow Transplant	Bone Marrow Transplant	Hematology	Medical Specialties
Pediatric Bone Marrow Transplant	Bone Marrow Transplant	Hematology	Medical Specialties
Hematology	Hematology	Hematology	Medical Specialties
Hematology And Oncology	Hematology	Hematology	Medical Specialties
Pediatric Hematology	Pediatric Hematology	Hematology	Medical Specialties
Pediatric Hematology And Oncology	Pediatric Hematology	Hematology	Medical Specialties
Blood Banking Transfusion Medicine	Transfusion Medicine	Hematology	Medical Specialties
Infusion And Transfusion	Transfusion Medicine	Hematology	Medical Specialties
Transfusion Medicine	Transfusion Medicine	Hematology	Medical Specialties
Infection Control	Infectious Disease	Infectious Disease	Medical Specialties
Infectious Disease	Infectious Disease	Infectious Disease	Medical Specialties
Infectious Diseases	Infectious Disease	Infectious Disease	Medical Specialties

Pediatric Infectious Disease	Pediatric Infectious Disease	Infectious Disease	Medical Specialties
Dialysis	Dialysis	Nephrology	Medical Specialties
Nephrology	Nephrology	Nephrology	Medical Specialties
Pediatric Nephrology	Pediatric Nephrology	Nephrology	Medical Specialties
Gynecologic Oncology	Gynecological Oncology	Oncology	Medical Specialties
Gynecologic Oncology Cancer Center	Gynecological Oncology	Oncology	Medical Specialties
Gynecological Oncology	Gynecological Oncology	Oncology	Medical Specialties
Medical Oncology	Medical Oncology	Oncology	Medical Specialties
Neuro Oncology	Neuro Oncology	Oncology	Medical Specialties
Cancer Center	Oncology	Oncology	Medical Specialties
Cancer Risk Program Cancer Center	Oncology	Oncology	Medical Specialties
Dental Oncology	Oncology	Oncology	Medical Specialties
Gastrointestinal Oncology	Oncology	Oncology	Medical Specialties
Genetics Cancer Center	Oncology	Oncology	Medical Specialties
Head And Neck Cancer Center	Oncology	Oncology	Medical Specialties
Hematology And Oncology	Oncology	Oncology	Medical Specialties
Oncology	Oncology	Oncology	Medical Specialties
Psychology Cancer Center	Oncology	Oncology	Medical Specialties
Support Service Cancer Center	Oncology	Oncology	Medical Specialties
Thoracic Oncology	Oncology	Oncology	Medical Specialties
Thoracic Oncology Cancer Center	Oncology	Oncology	Medical Specialties
Orthopedic Oncology	Orthopedic Oncology	Oncology	Medical Specialties
Orthopedic Surgical Oncology	Orthopedic Oncology	Oncology	Medical Specialties
Pediatric Hematology And Oncology	Pediatric Oncology	Oncology	Medical Specialties
Pediatric Oncology	Pediatric Oncology	Oncology	Medical Specialties
Radiation Oncology	Radiation Oncology	Oncology	Medical Specialties
Radiation Therapy	Radiation Oncology	Oncology	Medical Specialties
Cancer Survivorship	Surgical Oncology	Oncology	Medical Specialties
Orthopedic Surgical Oncology	Surgical Oncology	Oncology	Medical Specialties
Surgical Oncology	Surgical Oncology	Oncology	Medical Specialties
Urologic Oncology	Urologic Oncology	Oncology	Medical Specialties
Hospice And Palliative Medicine	Palliative Care	Palliative Care	Medical Specialties
Hospice Services	Palliative Care	Palliative Care	Medical Specialties
Palliative	Palliative Care	Palliative Care	Medical Specialties
Palliative Care	Palliative Care	Palliative Care	Medical Specialties
Palliative Care Home	Palliative Care	Palliative Care	Medical Specialties
Palliative Care Medicine	Palliative Care	Palliative Care	Medical Specialties
Palliative Medicine	Palliative Care	Palliative Care	Medical Specialties

Preventative Medicine	Prophylaxic Medicine	Prophylaxic Medicine	Medical Specialties
Pediatric Pulmonary Function	Pediatric Pulmonology	Pulmonology	Medical Specialties
Pediatric Pulmonology	Pediatric Pulmonology	Pulmonology	Medical Specialties
Pulmonary Function And Bronchoscopy	Pulmonology	Pulmonology	Medical Specialties
Pulmonology	Pulmonology	Pulmonology	Medical Specialties
Reproductive Endocrinology	Reproductive Endocrinology	Reproductive Endocrinology	Medical Specialties
Reproductive Endocrinology And Infertility	Reproductive Endocrinology	Reproductive Endocrinology	Medical Specialties
Pediatric Rheumatology	Pediatric Rheumatology	Rheumatology	Medical Specialties
Rheumatology	Rheumatology	Rheumatology	Medical Specialties
Pediatric Sleep Medicine	Sleep Medicine	Sleep Medicine	Medical Specialties
Sleep Medicine	Sleep Medicine	Sleep Medicine	Medical Specialties
Eeg	EEG	Neurology	Neurology and Psychiatry
Neurodevelopmental Disabilities	Neurodevelopmental Disabilities	Neurology	Neurology and Psychiatry
Neurodevelopmental Disability	Neurodevelopmental Disabilities	Neurology	Neurology and Psychiatry
Neuro Interventional Radiology	Neurology	Neurology	Neurology and Psychiatry
Neuro Ophthalmology	Neurology	Neurology	Neurology and Psychiatry
Neurodiagnostics	Neurology	Neurology	Neurology and Psychiatry
Neurology	Neurology	Neurology	Neurology and Psychiatry
Neuromuscular Medicine	Neurology	Neurology	Neurology and Psychiatry
Neuromusculoskeletal Medicine	Neurology	Neurology	Neurology and Psychiatry
Neuropsychology	Neurology	Neurology	Neurology and Psychiatry
Neuroradiology	Neurology	Neurology	Neurology and Psychiatry
Psychiatry And Neurology	Neurology	Neurology	Neurology and Psychiatry
Pediatric Neurology	Pediatric Neurology	Neurology	Neurology and Psychiatry
Addiction Medicine	Addiction Medicine	Psychiatry	Neurology and Psychiatry
Behavioral Health	Developmental and Behavioral Health	Psychiatry	Neurology and Psychiatry
Developmental And Behavioral Pediatrics	Developmental and Behavioral Health	Psychiatry	Neurology and Psychiatry
Neurodevelopmental Disabilities	Developmental and Behavioral Health	Psychiatry	Neurology and Psychiatry
Neurodevelopmental Disability	Developmental and Behavioral Health	Psychiatry	Neurology and Psychiatry
Mental Health	Mental Health	Psychiatry	Neurology and Psychiatry
Child And Adolescent Psychiatry	Pediatric Psychiatry	Psychiatry	Neurology and Psychiatry
Pediatric Psychiatry	Pediatric Psychiatry	Psychiatry	Neurology and Psychiatry
Pediatric Psychology	Pediatric Psychology	Psychiatry	Neurology and Psychiatry
Psychiatry	Psychiatry	Psychiatry	Neurology and Psychiatry
Psychiatry And Neurology	Psychiatry	Psychiatry	Neurology and Psychiatry
Neuropsychology	Psychology	Psychiatry	Neurology and Psychiatry

Psychology	Psychology	Psychiatry	Neurology and Psychiatry
Psychology Cancer Center	Psychology	Psychiatry	Neurology and Psychiatry
Gynecology	Gynecology	Obstetrics and Gynecology	Obstetrics and Gynecology
Maternal And Fetal Medicine	Maternal Fetal Medicine	Obstetrics and Gynecology	Obstetrics and Gynecology
Maternal Fetal Medicine	Maternal Fetal Medicine	Obstetrics and Gynecology	Obstetrics and Gynecology
Certified Nurse Midwife	Midwife	Obstetrics and Gynecology	Obstetrics and Gynecology
Midwife	Midwife	Obstetrics and Gynecology	Obstetrics and Gynecology
Midwifery	Midwife	Obstetrics and Gynecology	Obstetrics and Gynecology
Obstetrics	Obstetrics	Obstetrics and Gynecology	Obstetrics and Gynecology
Obstetrics And Gynecology	Obstetrics and Gynecology	Obstetrics and Gynecology	Obstetrics and Gynecology
Female Pelvic And Reconstructive Surgery	Pelvic Medicine and Reconstructive Surgery	Obstetrics and Gynecology	Obstetrics and Gynecology
Pelvic Medicine And Reconstructive Surgery	Pelvic Medicine and Reconstructive Surgery	Obstetrics and Gynecology	Obstetrics and Gynecology
Perinatology	Perinatology	Obstetrics and Gynecology	Obstetrics and Gynecology
Urogynecology	Urogynecology	Obstetrics and Gynecology	Obstetrics and Gynecology
Breast Care Cancer Center	Breast Health	Womens Health	Obstetrics and Gynecology
Breast Health	Breast Health	Womens Health	Obstetrics and Gynecology
Lactation	Lactation Services	Womens Health	Obstetrics and Gynecology
Lactation Services	Lactation Services	Womens Health	Obstetrics and Gynecology
Women S Health	Womens Health	Womens Health	Obstetrics and Gynecology
Womens Health	Womens Health	Womens Health	Obstetrics and Gynecology
Neuro Ophthalmology	Neuro-Ophthalmology	Ophthalmology	Ophthalmology
Oculoplastics Ophthalmology	Oculoplastics Ophthalmology	Ophthalmology	Ophthalmology
Ophthalmology	Ophthalmology	Ophthalmology	Ophthalmology
Pediatric Ophthalmology	Pediatric Ophthalmology	Ophthalmology	Ophthalmology
Optometrist Tlg	Optometry	Optometry	Ophthalmology
Optometry	Optometry	Optometry	Ophthalmology
Als	Other Specialties	Other Specialties	Other Specialties
Antimicrobial Stewardship	Other Specialties	Other Specialties	Other Specialties
Bcho	Other Specialties	Other Specialties	Other Specialties
Care At Home	Other Specialties	Other Specialties	Other Specialties
Case Management	Other Specialties	Other Specialties	Other Specialties
Clinical Research	Other Specialties	Other Specialties	Other Specialties
Dysphagia	Other Specialties	Other Specialties	Other Specialties
Dysplasia	Other Specialties	Other Specialties	Other Specialties
Epiccare Link	Other Specialties	Other Specialties	Other Specialties

Epilepsy	Other Specialties	Other Specialties	Other Specialties
Ethics	Other Specialties	Other Specialties	Other Specialties
Family Medicine	Other Specialties	Other Specialties	Other Specialties
Home Health Services	Other Specialties	Other Specialties	Other Specialties
Initial Dept	Other Specialties	Other Specialties	Other Specialties
Interpreting Services	Other Specialties	Other Specialties	Other Specialties
Investigational Therapy	Other Specialties	Other Specialties	Other Specialties
Iron Overload	Other Specialties	Other Specialties	Other Specialties
Marriage Family Therapist	Other Specialties	Other Specialties	Other Specialties
Medical Specialties	Other Specialties	Other Specialties	Other Specialties
Memory And Aging	Other Specialties	Other Specialties	Other Specialties
Multidisciplinary Conference	Other Specialties	Other Specialties	Other Specialties
Nephrology	Other Specialties	Other Specialties	Other Specialties
Nurse	Other Specialties	Other Specialties	Other Specialties
Nurse Practitioner	Other Specialties	Other Specialties	Other Specialties
Oncology	Other Specialties	Other Specialties	Other Specialties
Other	Other Specialties	Other Specialties	Other Specialties
Other Specialties	Other Specialties	Other Specialties	Other Specialties
Pcp	Other Specialties	Other Specialties	Other Specialties
Pelvic Physiology	Other Specialties	Other Specialties	Other Specialties
Physician	Other Specialties	Other Specialties	Other Specialties
Physician Assistant	Other Specialties	Other Specialties	Other Specialties
Post Discharge Phone Call	Other Specialties	Other Specialties	Other Specialties
Prenatal Diagnosis	Other Specialties	Other Specialties	Other Specialties
Quality Improvement	Other Specialties	Other Specialties	Other Specialties
Registered Nurse First Assistant	Other Specialties	Other Specialties	Other Specialties
Rett Syndrome	Other Specialties	Other Specialties	Other Specialties
Social Services	Other Specialties	Other Specialties	Other Specialties
Social Work	Other Specialties	Other Specialties	Other Specialties
Social Worker	Other Specialties	Other Specialties	Other Specialties
Spiritual Care	Other Specialties	Other Specialties	Other Specialties
Symptom Management	Other Specialties	Other Specialties	Other Specialties
Therapist	Other Specialties	Other Specialties	Other Specialties
Transfer Center	Other Specialties	Other Specialties	Other Specialties
Ucsf	Other Specialties	Other Specialties	Other Specialties
Occupational Medicine	Occupational Medicine	Occupational Medicine	Physical Medicine and Rehabilitation
Occupational Therapy	Occupational Medicine	Occupational Medicine	Physical Medicine and Rehabilitation
Pediatric Occupational Therapy	Pediatric Occupational Medicine	Occupational Medicine	Physical Medicine and Rehabilitation

Pain Medicine	Pain Medicine	Pain Medicine	Physical Medicine and Rehabilitation
Pediatric Physical Medicine And Rehabilitation	Pediatric Physical Medicine	Physical Medicine	Physical Medicine and Rehabilitation
Pediatric Physical Therapy	Pediatric Physical Medicine	Physical Medicine	Physical Medicine and Rehabilitation
Pediatric Rehabilitation	Pediatric Physical Medicine	Physical Medicine	Physical Medicine and Rehabilitation
Cardiac Rehabilitation	Physical Medicine and Rehabilitation	Physical Medicine	Physical Medicine and Rehabilitation
Physical Medicine	Physical Medicine and Rehabilitation	Physical Medicine	Physical Medicine and Rehabilitation
Physical Medicine And Rehabilitation	Physical Medicine and Rehabilitation	Physical Medicine	Physical Medicine and Rehabilitation
Rehabilitation	Physical Medicine and Rehabilitation	Physical Medicine	Physical Medicine and Rehabilitation
Physical Therapy	Physical Therapy	Physical Medicine	Physical Medicine and Rehabilitation
Respiratory Therapy	Respiratory Therapy	Physical Medicine	Physical Medicine and Rehabilitation
Speech Language Pathology	Speech Language Pathology	Speech Language Pathology	Physical Medicine and Rehabilitation
Speech Pathology	Speech Language Pathology	Speech Language Pathology	Physical Medicine and Rehabilitation
Pediatric Speech Therapy	Speech Therapy	Speech Language Pathology	Physical Medicine and Rehabilitation
Speech Therapy	Speech Therapy	Speech Language Pathology	Physical Medicine and Rehabilitation
Exercise	Sports Medicine	Sports Medicine	Physical Medicine and Rehabilitation
Sports Medicine	Sports Medicine	Sports Medicine	Physical Medicine and Rehabilitation
Acupuncture	Acupuncture	Alternative Medicine	Primary Care
Alternative Medicine	Alternative Medicine	Alternative Medicine	Primary Care
Holistic Medicine	Holistic Medicine	Alternative Medicine	Primary Care
Integrative Medicine	Holistic Medicine	Alternative Medicine	Primary Care
Massage Therapy	Massage Therapy	Alternative Medicine	Primary Care
Neuromuscular Medicine	Neuromusculoskeletal Medicine	Alternative Medicine	Primary Care
Neuromusculoskeletal Medicine	Neuromusculoskeletal Medicine	Alternative Medicine	Primary Care
Diet	Diet	Diet and Nutrition	Primary Care
Dietician	Diet	Diet and Nutrition	Primary Care
Weight Management	Diet	Diet and Nutrition	Primary Care
Nutrition	Nutrition	Diet and Nutrition	Primary Care
Employee Health Services	Family Medicine	Family Medicine	Primary Care
Executive Health	Family Medicine	Family Medicine	Primary Care
Family Medicine	Family Medicine	Family Medicine	Primary Care
General Practice	Family Medicine	Family Medicine	Primary Care
Primary Care	Family Medicine	Family Medicine	Primary Care
General Internal Medicine	General Internal Medicine	General Internal Medicine	Primary Care

Internal Medicine	General Internal Medicine	General Internal Medicine	Primary Care
Infusion	Infusion	General Internal Medicine	Primary Care
Infusion And Transfusion	Infusion	General Internal Medicine	Primary Care
Pediatric Infusion And Transfusion	Infusion	General Internal Medicine	Primary Care
Adolescent Medicine	Adolescent Medicine	General Pediatrics	Primary Care
Center For Child Protection	General Pediatrics	General Pediatrics	Primary Care
Child Life Services	General Pediatrics	General Pediatrics	Primary Care
General Pediatrics	General Pediatrics	General Pediatrics	Primary Care
Pediatric Gender Care	General Pediatrics	General Pediatrics	Primary Care
Pediatric Transport	General Pediatrics	General Pediatrics	Primary Care
Pediatrics	General Pediatrics	General Pediatrics	Primary Care
Pediatrics Specialties	General Pediatrics	General Pediatrics	Primary Care
Neonatology	Perinatal and Neonatology	General Pediatrics	Primary Care
Geriatric Care	Geriatric Medicine	Geriatric Medicine	Primary Care
Geriatric Medicine	Geriatric Medicine	Geriatric Medicine	Primary Care
Home Health	Home Health	Home Health	Sub-Acute Care Medicine
Home Health Services	Home Health	Home Health	Sub-Acute Care Medicine
Cardiac Surgery	Cardiac Surgery	Cardiothoracic Surgery	Surgical Specialties
Cardiothoracic Surgery	Cardiothoracic Surgery	Cardiothoracic Surgery	Surgical Specialties
Electrophysiology	Electrophysiology	Cardiothoracic Surgery	Surgical Specialties
Interventional Cardiology	Interventional Cardiology	Cardiothoracic Surgery	Surgical Specialties
Pediatric Cardiothoracic Surgery	Pediatric Cardiothoracic Surgery	Cardiothoracic Surgery	Surgical Specialties
Thoracic Oncology	Thoracic Surgery	Cardiothoracic Surgery	Surgical Specialties
Thoracic Oncology Cancer Center	Thoracic Surgery	Cardiothoracic Surgery	Surgical Specialties
Thoracic Surgery	Thoracic Surgery	Cardiothoracic Surgery	Surgical Specialties
Colon And Rectal Surgery	Colorectal Surgery	Colorectal Surgery	Surgical Specialties
Colorectal Surgery	Colorectal Surgery	Colorectal Surgery	Surgical Specialties
Breast Surgery	Breast Surgery	General Surgery	Surgical Specialties
Gastrointestinal Surgery	General Surgery	General Surgery	Surgical Specialties
General Surgery	General Surgery	General Surgery	Surgical Specialties
Periop	General Surgery	General Surgery	Surgical Specialties
Pre Admission Testing	General Surgery	General Surgery	Surgical Specialties
Surgical Specialties	General Surgery	General Surgery	Surgical Specialties
Pediatric General Surgery	Pediatric General Surgery	General Surgery	Surgical Specialties
Pediatric Surgery	Pediatric General Surgery	General Surgery	Surgical Specialties
Neurosurgery	Neurosurgery	Neurosurgery	Surgical Specialties
Pediatric Neurosurgery	Pediatric Neurosurgery	Neurosurgery	Surgical Specialties

Hand Surgery	Hand Surgery	Orthopedics	Surgical Specialties
Orthopedic Surgery Hand	Hand Surgery	Orthopedics	Surgical Specialties
Orthopedic Surgery	Orthopedics	Orthopedics	Surgical Specialties
Orthopedic Surgery Arthroplasty	Orthopedics	Orthopedics	Surgical Specialties
Orthopedic Surgery Foot And Ankle	Orthopedics	Orthopedics	Surgical Specialties
Orthopedic Surgery Sports	Orthopedics	Orthopedics	Surgical Specialties
Orthopedics	Orthopedics	Orthopedics	Surgical Specialties
Orthotics	Orthopedics	Orthopedics	Surgical Specialties
Pediatric Orthopedic Surgery	Pediatric Orthopedics	Orthopedics	Surgical Specialties
Pediatric Orthopedics	Pediatric Orthopedics	Orthopedics	Surgical Specialties
Orthopedic Surgery Spine	Spine Surgery	Orthopedics	Surgical Specialties
Spine Surgery	Spine Surgery	Orthopedics	Surgical Specialties
Audiology	Audiology	Otorhinolaryngology	Surgical Specialties
Head And Neck Surgery	Head and Neck Surgery	Otorhinolaryngology	Surgical Specialties
Otolaryngology Head And Neck Surgery	Head and Neck Surgery	Otorhinolaryngology	Surgical Specialties
Pediatric Otolaryngology Head And Neck Surgery	Head and Neck Surgery	Otorhinolaryngology	Surgical Specialties
Otolaryngology	Otorhinolaryngology	Otorhinolaryngology	Surgical Specialties
Otorhinolaryngology	Otorhinolaryngology	Otorhinolaryngology	Surgical Specialties
Pulmonary Function And Bronchoscopy	Otorhinolaryngology	Otorhinolaryngology	Surgical Specialties
Pediatric Otolaryngology	Pediatric Otolaryngology	Otorhinolaryngology	Surgical Specialties
Pediatric Otolaryngology Head And Neck Surgery	Pediatric Otolaryngology	Otorhinolaryngology	Surgical Specialties
Pediatric Plastic Surgery	Pediatric Plastic Surgery	Plastic Surgery	Surgical Specialties
Craniofacial Anomalies	Plastic Surgery	Plastic Surgery	Surgical Specialties
Ohns Plastics	Plastic Surgery	Plastic Surgery	Surgical Specialties
Plastic Surgery	Plastic Surgery	Plastic Surgery	Surgical Specialties
Podiatry	Podiatry	Podiatry	Surgical Specialties
Endocrine Surgery	Reproductive and Endocrine Surgery	Reproductive and Endocrine Surgery	Surgical Specialties
Pediatric Transplant Hepatology	Pediatric Transplant Hepatology	Transplant Surgery	Surgical Specialties
Liver Transplant	Transplant Hepatology	Transplant Surgery	Surgical Specialties
Transplant Hepatology	Transplant Hepatology	Transplant Surgery	Surgical Specialties
Advanced Heart Failure Transplant Cardiology	Transplant Surgery	Transplant Surgery	Surgical Specialties
Heart Transplant	Transplant Surgery	Transplant Surgery	Surgical Specialties
Kidney Transplantation	Transplant Surgery	Transplant Surgery	Surgical Specialties
Lung Transplant	Transplant Surgery	Transplant Surgery	Surgical Specialties
Pancreas Transplant	Transplant Surgery	Transplant Surgery	Surgical Specialties
Transplant	Transplant Surgery	Transplant Surgery	Surgical Specialties

Transplant Surgery	Transplant Surgery	Transplant Surgery	Surgical Specialties
Trauma Surgery	Trauma Surgery	Trauma Surgery	Surgical Specialties
Pediatric Urology	Pediatric Urology	Urology	Surgical Specialties
Urology	Urology	Urology	Surgical Specialties
Vascular And Interventional Radiology	Vascular Surgery	Vascular Surgery	Surgical Specialties
Vascular Surg	Vascular Surgery	Vascular Surgery	Surgical Specialties
Vascular Surgery	Vascular Surgery	Vascular Surgery	Surgical Specialties
Wound Care	Wound Medicine	Wound Medicine	Surgical Specialties

Supplementary Table 2. Oncology Physician EHR Use over Time

Oncology Sub-Specialty	Month	EHR Time Variable	EHR Minutes per Week (Mean)
1 Medical	7/28/19	a. Total Weekly Active EHR Time	507.59
1 Medical	9/1/19	a. Total Weekly Active EHR Time	507.67
1 Medical	9/29/19	a. Total Weekly Active EHR Time	510.16
1 Medical	10/27/19	a. Total Weekly Active EHR Time	504.67
1 Medical	12/1/19	a. Total Weekly Active EHR Time	481.56
1 Medical	12/29/19	a. Total Weekly Active EHR Time	499.60
1 Medical	1/26/20	a. Total Weekly Active EHR Time	526.86
1 Medical	3/1/20	a. Total Weekly Active EHR Time	505.51
1 Medical	3/29/20	a. Total Weekly Active EHR Time	500.63
1 Medical	4/26/20	a. Total Weekly Active EHR Time	527.78
1 Medical	5/31/20	a. Total Weekly Active EHR Time	559.71
1 Medical	6/28/20	a. Total Weekly Active EHR Time	539.99
1 Medical	7/26/20	a. Total Weekly Active EHR Time	542.86
1 Medical	8/30/20	a. Total Weekly Active EHR Time	544.38
1 Medical	9/27/20	a. Total Weekly Active EHR Time	546.02
1 Medical	11/1/20	a. Total Weekly Active EHR Time	535.17
1 Medical	11/29/20	a. Total Weekly Active EHR Time	535.76
1 Medical	12/27/20	a. Total Weekly Active EHR Time	547.46
1 Medical	1/31/21	a. Total Weekly Active EHR Time	579.80
1 Medical	2/28/21	a. Total Weekly Active EHR Time	589.24
1 Medical	3/28/21	a. Total Weekly Active EHR Time	568.34
1 Medical	4/25/21	a. Total Weekly Active EHR Time	579.83
1 Medical	5/30/21	a. Total Weekly Active EHR Time	549.44
1 Medical	6/27/21	a. Total Weekly Active EHR Time	545.24
1 Medical	8/1/21	a. Total Weekly Active EHR Time	564.60
1 Medical	8/29/21	a. Total Weekly Active EHR Time	563.36
1 Medical	9/26/21	a. Total Weekly Active EHR Time	576.88
1 Medical	10/31/21	a. Total Weekly Active EHR Time	539.82
1 Medical	11/28/21	a. Total Weekly Active EHR Time	557.95
1 Medical	12/26/21	a. Total Weekly Active EHR Time	543.29
1 Medical	1/30/22	a. Total Weekly Active EHR Time	580.21
1 Medical	2/27/22	a. Total Weekly Active EHR Time	586.40
1 Medical	3/27/22	a. Total Weekly Active EHR Time	575.48
2 Pediatric	7/28/19	a. Total Weekly Active EHR Time	229.38
2 Pediatric	9/1/19	a. Total Weekly Active EHR Time	214.80
2 Pediatric	9/29/19	a. Total Weekly Active EHR Time	230.00
2 Pediatric	10/27/19	a. Total Weekly Active EHR Time	218.01
2 Pediatric	12/1/19	a. Total Weekly Active EHR Time	209.48
2 Pediatric	12/29/19	a. Total Weekly Active EHR Time	218.37
2 Pediatric	1/26/20	a. Total Weekly Active EHR Time	232.58
2 Pediatric	3/1/20	a. Total Weekly Active EHR Time	210.06
2 Pediatric	3/29/20	a. Total Weekly Active EHR Time	204.07
2 Pediatric	4/26/20	a. Total Weekly Active EHR Time	217.39

2 Pediatric	5/31/20	a. Total Weekly Active EHR Time		238.05
2 Pediatric	6/28/20	a. Total Weekly Active EHR Time		227.49
2 Pediatric	7/26/20	a. Total Weekly Active EHR Time		235.75
2 Pediatric	8/30/20	a. Total Weekly Active EHR Time		225.48
2 Pediatric	9/27/20	a. Total Weekly Active EHR Time		237.03
2 Pediatric	11/1/20	a. Total Weekly Active EHR Time		230.18
2 Pediatric	11/29/20	a. Total Weekly Active EHR Time		230.27
2 Pediatric	12/27/20	a. Total Weekly Active EHR Time		236.44
2 Pediatric	1/31/21	a. Total Weekly Active EHR Time		247.60
2 Pediatric	2/28/21	a. Total Weekly Active EHR Time		265.59
2 Pediatric	3/28/21	a. Total Weekly Active EHR Time		250.92
2 Pediatric	4/25/21	a. Total Weekly Active EHR Time		256.59
2 Pediatric	5/30/21	a. Total Weekly Active EHR Time		243.31
2 Pediatric	6/27/21	a. Total Weekly Active EHR Time		245.40
2 Pediatric	8/1/21	a. Total Weekly Active EHR Time		258.46
2 Pediatric	8/29/21	a. Total Weekly Active EHR Time		246.21
2 Pediatric	9/26/21	a. Total Weekly Active EHR Time		252.03
2 Pediatric	10/31/21	a. Total Weekly Active EHR Time		243.84
2 Pediatric	11/28/21	a. Total Weekly Active EHR Time		246.26
2 Pediatric	12/26/21	a. Total Weekly Active EHR Time		240.60
2 Pediatric	1/30/22	a. Total Weekly Active EHR Time		265.05
2 Pediatric	2/27/22	a. Total Weekly Active EHR Time		265.55
2 Pediatric	3/27/22	a. Total Weekly Active EHR Time		259.64
3 Radiation	7/28/19	a. Total Weekly Active EHR Time		245.38
3 Radiation	9/1/19	a. Total Weekly Active EHR Time		243.43
3 Radiation	9/29/19	a. Total Weekly Active EHR Time		254.44
3 Radiation	10/27/19	a. Total Weekly Active EHR Time		237.46
3 Radiation	12/1/19	a. Total Weekly Active EHR Time		237.08
3 Radiation	12/29/19	a. Total Weekly Active EHR Time		238.82
3 Radiation	1/26/20	a. Total Weekly Active EHR Time		254.39
3 Radiation	3/1/20	a. Total Weekly Active EHR Time		249.42
3 Radiation	3/29/20	a. Total Weekly Active EHR Time		248.23
3 Radiation	4/26/20	a. Total Weekly Active EHR Time		250.65
3 Radiation	5/31/20	a. Total Weekly Active EHR Time		264.98
3 Radiation	6/28/20	a. Total Weekly Active EHR Time		253.32
3 Radiation	7/26/20	a. Total Weekly Active EHR Time		250.74
3 Radiation	8/30/20	a. Total Weekly Active EHR Time		255.35
3 Radiation	9/27/20	a. Total Weekly Active EHR Time		255.48
3 Radiation	11/1/20	a. Total Weekly Active EHR Time		249.45
3 Radiation	11/29/20	a. Total Weekly Active EHR Time		252.88
3 Radiation	12/27/20	a. Total Weekly Active EHR Time		257.57
3 Radiation	1/31/21	a. Total Weekly Active EHR Time		268.63
3 Radiation	2/28/21	a. Total Weekly Active EHR Time		277.94
3 Radiation	3/28/21	a. Total Weekly Active EHR Time		274.21
3 Radiation	4/25/21	a. Total Weekly Active EHR Time		272.48
3 Radiation	5/30/21	a. Total Weekly Active EHR Time		264.76

3 Radiation	6/27/21	a. Total Weekly Active EHR Time		262.65
3 Radiation	8/1/21	a. Total Weekly Active EHR Time		270.88
3 Radiation	8/29/21	a. Total Weekly Active EHR Time		263.71
3 Radiation	9/26/21	a. Total Weekly Active EHR Time		276.79
3 Radiation	10/31/21	a. Total Weekly Active EHR Time		261.41
3 Radiation	11/28/21	a. Total Weekly Active EHR Time		270.51
3 Radiation	12/26/21	a. Total Weekly Active EHR Time		263.44
3 Radiation	1/30/22	a. Total Weekly Active EHR Time		278.01
3 Radiation	2/27/22	a. Total Weekly Active EHR Time		282.24
3 Radiation	3/27/22	a. Total Weekly Active EHR Time		275.29
4 Gynecology	7/28/19	a. Total Weekly Active EHR Time		287.42
4 Gynecology	9/1/19	a. Total Weekly Active EHR Time		287.58
4 Gynecology	9/29/19	a. Total Weekly Active EHR Time		300.52
4 Gynecology	10/27/19	a. Total Weekly Active EHR Time		288.22
4 Gynecology	12/1/19	a. Total Weekly Active EHR Time		274.33
4 Gynecology	12/29/19	a. Total Weekly Active EHR Time		280.96
4 Gynecology	1/26/20	a. Total Weekly Active EHR Time		301.63
4 Gynecology	3/1/20	a. Total Weekly Active EHR Time		304.32
4 Gynecology	3/29/20	a. Total Weekly Active EHR Time		279.15
4 Gynecology	4/26/20	a. Total Weekly Active EHR Time		292.92
4 Gynecology	5/31/20	a. Total Weekly Active EHR Time		319.75
4 Gynecology	6/28/20	a. Total Weekly Active EHR Time		307.11
4 Gynecology	7/26/20	a. Total Weekly Active EHR Time		306.43
4 Gynecology	8/30/20	a. Total Weekly Active EHR Time		309.51
4 Gynecology	9/27/20	a. Total Weekly Active EHR Time		321.59
4 Gynecology	11/1/20	a. Total Weekly Active EHR Time		301.23
4 Gynecology	11/29/20	a. Total Weekly Active EHR Time		304.97
4 Gynecology	12/27/20	a. Total Weekly Active EHR Time		307.95
4 Gynecology	1/31/21	a. Total Weekly Active EHR Time		318.42
4 Gynecology	2/28/21	a. Total Weekly Active EHR Time		329.35
4 Gynecology	3/28/21	a. Total Weekly Active EHR Time		314.28
4 Gynecology	4/25/21	a. Total Weekly Active EHR Time		326.69
4 Gynecology	5/30/21	a. Total Weekly Active EHR Time		304.98
4 Gynecology	6/27/21	a. Total Weekly Active EHR Time		303.26
4 Gynecology	8/1/21	a. Total Weekly Active EHR Time		313.18
4 Gynecology	8/29/21	a. Total Weekly Active EHR Time		305.79
4 Gynecology	9/26/21	a. Total Weekly Active EHR Time		327.81
4 Gynecology	10/31/21	a. Total Weekly Active EHR Time		300.40
4 Gynecology	11/28/21	a. Total Weekly Active EHR Time		305.95
4 Gynecology	12/26/21	a. Total Weekly Active EHR Time		300.61
4 Gynecology	1/30/22	a. Total Weekly Active EHR Time		322.74
4 Gynecology	2/27/22	a. Total Weekly Active EHR Time		316.02
4 Gynecology	3/27/22	a. Total Weekly Active EHR Time		326.39
6 Surgical	7/28/19	a. Total Weekly Active EHR Time		255.80
6 Surgical	9/1/19	a. Total Weekly Active EHR Time		248.50
6 Surgical	9/29/19	a. Total Weekly Active EHR Time		256.19

6 Surgical	10/27/19	a. Total Weekly Active EHR Time		239.67
6 Surgical	12/1/19	a. Total Weekly Active EHR Time		229.21
6 Surgical	12/29/19	a. Total Weekly Active EHR Time		242.69
6 Surgical	1/26/20	a. Total Weekly Active EHR Time		257.45
6 Surgical	3/1/20	a. Total Weekly Active EHR Time		247.17
6 Surgical	3/29/20	a. Total Weekly Active EHR Time		200.22
6 Surgical	4/26/20	a. Total Weekly Active EHR Time		217.51
6 Surgical	5/31/20	a. Total Weekly Active EHR Time		260.80
6 Surgical	6/28/20	a. Total Weekly Active EHR Time		257.15
6 Surgical	7/26/20	a. Total Weekly Active EHR Time		267.44
6 Surgical	8/30/20	a. Total Weekly Active EHR Time		263.90
6 Surgical	9/27/20	a. Total Weekly Active EHR Time		265.40
6 Surgical	11/1/20	a. Total Weekly Active EHR Time		250.83
6 Surgical	11/29/20	a. Total Weekly Active EHR Time		253.53
6 Surgical	12/27/20	a. Total Weekly Active EHR Time		248.97
6 Surgical	1/31/21	a. Total Weekly Active EHR Time		269.09
6 Surgical	2/28/21	a. Total Weekly Active EHR Time		272.20
6 Surgical	3/28/21	a. Total Weekly Active EHR Time		268.00
6 Surgical	4/25/21	a. Total Weekly Active EHR Time		272.51
6 Surgical	5/30/21	a. Total Weekly Active EHR Time		256.86
6 Surgical	6/27/21	a. Total Weekly Active EHR Time		256.27
6 Surgical	8/1/21	a. Total Weekly Active EHR Time		266.34
6 Surgical	8/29/21	a. Total Weekly Active EHR Time		262.53
6 Surgical	9/26/21	a. Total Weekly Active EHR Time		269.28
6 Surgical	10/31/21	a. Total Weekly Active EHR Time		253.66
6 Surgical	11/28/21	a. Total Weekly Active EHR Time		253.50
6 Surgical	12/26/21	a. Total Weekly Active EHR Time		248.22
6 Surgical	1/30/22	a. Total Weekly Active EHR Time		264.26
6 Surgical	2/27/22	a. Total Weekly Active EHR Time		256.74
6 Surgical	3/27/22	a. Total Weekly Active EHR Time		266.17
1 Medical	7/28/19	b. Active InBasket Time		59.26
1 Medical	9/1/19	b. Active InBasket Time		58.68
1 Medical	9/29/19	b. Active InBasket Time		59.85
1 Medical	10/27/19	b. Active InBasket Time		58.09
1 Medical	12/1/19	b. Active InBasket Time		56.39
1 Medical	12/29/19	b. Active InBasket Time		58.79
1 Medical	1/26/20	b. Active InBasket Time		61.39
1 Medical	3/1/20	b. Active InBasket Time		66.48
1 Medical	3/29/20	b. Active InBasket Time		67.14
1 Medical	4/26/20	b. Active InBasket Time		67.24
1 Medical	5/31/20	b. Active InBasket Time		69.21
1 Medical	6/28/20	b. Active InBasket Time		66.52
1 Medical	7/26/20	b. Active InBasket Time		66.24
1 Medical	8/30/20	b. Active InBasket Time		66.15
1 Medical	9/27/20	b. Active InBasket Time		66.10
1 Medical	11/1/20	b. Active InBasket Time		64.30

1 Medical	11/29/20	b. Active InBasket Time		65.39
1 Medical	12/27/20	b. Active InBasket Time		66.92
1 Medical	1/31/21	b. Active InBasket Time		71.33
1 Medical	2/28/21	b. Active InBasket Time		72.77
1 Medical	3/28/21	b. Active InBasket Time		71.40
1 Medical	4/25/21	b. Active InBasket Time		72.96
1 Medical	5/30/21	b. Active InBasket Time		69.50
1 Medical	6/27/21	b. Active InBasket Time		68.83
1 Medical	8/1/21	b. Active InBasket Time		72.16
1 Medical	8/29/21	b. Active InBasket Time		71.49
1 Medical	9/26/21	b. Active InBasket Time		73.04
1 Medical	10/31/21	b. Active InBasket Time		68.23
1 Medical	11/28/21	b. Active InBasket Time		71.27
1 Medical	12/26/21	b. Active InBasket Time		71.33
1 Medical	1/30/22	b. Active InBasket Time		73.99
1 Medical	2/27/22	b. Active InBasket Time		74.15
1 Medical	3/27/22	b. Active InBasket Time		72.52
2 Pediatric	7/28/19	b. Active InBasket Time		24.97
2 Pediatric	9/1/19	b. Active InBasket Time		23.37
2 Pediatric	9/29/19	b. Active InBasket Time		24.00
2 Pediatric	10/27/19	b. Active InBasket Time		22.12
2 Pediatric	12/1/19	b. Active InBasket Time		21.40
2 Pediatric	12/29/19	b. Active InBasket Time		21.71
2 Pediatric	1/26/20	b. Active InBasket Time		23.92
2 Pediatric	3/1/20	b. Active InBasket Time		23.03
2 Pediatric	3/29/20	b. Active InBasket Time		22.63
2 Pediatric	4/26/20	b. Active InBasket Time		22.76
2 Pediatric	5/31/20	b. Active InBasket Time		25.62
2 Pediatric	6/28/20	b. Active InBasket Time		24.47
2 Pediatric	7/26/20	b. Active InBasket Time		25.68
2 Pediatric	8/30/20	b. Active InBasket Time		24.36
2 Pediatric	9/27/20	b. Active InBasket Time		24.65
2 Pediatric	11/1/20	b. Active InBasket Time		24.30
2 Pediatric	11/29/20	b. Active InBasket Time		24.92
2 Pediatric	12/27/20	b. Active InBasket Time		25.23
2 Pediatric	1/31/21	b. Active InBasket Time		26.99
2 Pediatric	2/28/21	b. Active InBasket Time		30.57
2 Pediatric	3/28/21	b. Active InBasket Time		30.18
2 Pediatric	4/25/21	b. Active InBasket Time		30.06
2 Pediatric	5/30/21	b. Active InBasket Time		29.23
2 Pediatric	6/27/21	b. Active InBasket Time		29.69
2 Pediatric	8/1/21	b. Active InBasket Time		31.88
2 Pediatric	8/29/21	b. Active InBasket Time		31.12
2 Pediatric	9/26/21	b. Active InBasket Time		31.05
2 Pediatric	10/31/21	b. Active InBasket Time		30.07
2 Pediatric	11/28/21	b. Active InBasket Time		29.75

2 Pediatric	12/26/21	b. Active InBasket Time		30.40
2 Pediatric	1/30/22	b. Active InBasket Time		32.67
2 Pediatric	2/27/22	b. Active InBasket Time		32.53
2 Pediatric	3/27/22	b. Active InBasket Time		32.50
3 Radiation	7/28/19	b. Active InBasket Time		22.44
3 Radiation	9/1/19	b. Active InBasket Time		22.01
3 Radiation	9/29/19	b. Active InBasket Time		23.39
3 Radiation	10/27/19	b. Active InBasket Time		22.05
3 Radiation	12/1/19	b. Active InBasket Time		21.68
3 Radiation	12/29/19	b. Active InBasket Time		21.61
3 Radiation	1/26/20	b. Active InBasket Time		23.13
3 Radiation	3/1/20	b. Active InBasket Time		25.44
3 Radiation	3/29/20	b. Active InBasket Time		26.21
3 Radiation	4/26/20	b. Active InBasket Time		25.54
3 Radiation	5/31/20	b. Active InBasket Time		25.90
3 Radiation	6/28/20	b. Active InBasket Time		24.88
3 Radiation	7/26/20	b. Active InBasket Time		24.68
3 Radiation	8/30/20	b. Active InBasket Time		24.52
3 Radiation	9/27/20	b. Active InBasket Time		24.30
3 Radiation	11/1/20	b. Active InBasket Time		23.40
3 Radiation	11/29/20	b. Active InBasket Time		23.79
3 Radiation	12/27/20	b. Active InBasket Time		24.64
3 Radiation	1/31/21	b. Active InBasket Time		26.46
3 Radiation	2/28/21	b. Active InBasket Time		27.21
3 Radiation	3/28/21	b. Active InBasket Time		26.87
3 Radiation	4/25/21	b. Active InBasket Time		26.98
3 Radiation	5/30/21	b. Active InBasket Time		25.78
3 Radiation	6/27/21	b. Active InBasket Time		25.66
3 Radiation	8/1/21	b. Active InBasket Time		26.72
3 Radiation	8/29/21	b. Active InBasket Time		26.16
3 Radiation	9/26/21	b. Active InBasket Time		27.14
3 Radiation	10/31/21	b. Active InBasket Time		25.85
3 Radiation	11/28/21	b. Active InBasket Time		26.67
3 Radiation	12/26/21	b. Active InBasket Time		26.20
3 Radiation	1/30/22	b. Active InBasket Time		27.54
3 Radiation	2/27/22	b. Active InBasket Time		27.44
3 Radiation	3/27/22	b. Active InBasket Time		26.83
4 Gynecology	7/28/19	b. Active InBasket Time		45.55
4 Gynecology	9/1/19	b. Active InBasket Time		44.96
4 Gynecology	9/29/19	b. Active InBasket Time		46.85
4 Gynecology	10/27/19	b. Active InBasket Time		44.76
4 Gynecology	12/1/19	b. Active InBasket Time		42.30
4 Gynecology	12/29/19	b. Active InBasket Time		43.40
4 Gynecology	1/26/20	b. Active InBasket Time		46.59
4 Gynecology	3/1/20	b. Active InBasket Time		51.40
4 Gynecology	3/29/20	b. Active InBasket Time		46.03

4 Gynecology	4/26/20	b. Active InBasket Time	47.68
4 Gynecology	5/31/20	b. Active InBasket Time	50.76
4 Gynecology	6/28/20	b. Active InBasket Time	48.32
4 Gynecology	7/26/20	b. Active InBasket Time	48.76
4 Gynecology	8/30/20	b. Active InBasket Time	48.34
4 Gynecology	9/27/20	b. Active InBasket Time	50.78
4 Gynecology	11/1/20	b. Active InBasket Time	48.16
4 Gynecology	11/29/20	b. Active InBasket Time	48.51
4 Gynecology	12/27/20	b. Active InBasket Time	48.24
4 Gynecology	1/31/21	b. Active InBasket Time	51.81
4 Gynecology	2/28/21	b. Active InBasket Time	54.38
4 Gynecology	3/28/21	b. Active InBasket Time	52.09
4 Gynecology	4/25/21	b. Active InBasket Time	54.63
4 Gynecology	5/30/21	b. Active InBasket Time	51.16
4 Gynecology	6/27/21	b. Active InBasket Time	50.98
4 Gynecology	8/1/21	b. Active InBasket Time	51.81
4 Gynecology	8/29/21	b. Active InBasket Time	51.03
4 Gynecology	9/26/21	b. Active InBasket Time	54.68
4 Gynecology	10/31/21	b. Active InBasket Time	50.66
4 Gynecology	11/28/21	b. Active InBasket Time	52.05
4 Gynecology	12/26/21	b. Active InBasket Time	51.19
4 Gynecology	1/30/22	b. Active InBasket Time	54.32
4 Gynecology	2/27/22	b. Active InBasket Time	53.97
4 Gynecology	3/27/22	b. Active InBasket Time	54.63
6 Surgical	7/28/19	b. Active InBasket Time	40.99
6 Surgical	9/1/19	b. Active InBasket Time	39.41
6 Surgical	9/29/19	b. Active InBasket Time	40.03
6 Surgical	10/27/19	b. Active InBasket Time	37.38
6 Surgical	12/1/19	b. Active InBasket Time	35.79
6 Surgical	12/29/19	b. Active InBasket Time	37.77
6 Surgical	1/26/20	b. Active InBasket Time	41.04
6 Surgical	3/1/20	b. Active InBasket Time	41.89
6 Surgical	3/29/20	b. Active InBasket Time	35.49
6 Surgical	4/26/20	b. Active InBasket Time	37.28
6 Surgical	5/31/20	b. Active InBasket Time	42.41
6 Surgical	6/28/20	b. Active InBasket Time	41.04
6 Surgical	7/26/20	b. Active InBasket Time	43.74
6 Surgical	8/30/20	b. Active InBasket Time	42.31
6 Surgical	9/27/20	b. Active InBasket Time	43.34
6 Surgical	11/1/20	b. Active InBasket Time	40.49
6 Surgical	11/29/20	b. Active InBasket Time	40.19
6 Surgical	12/27/20	b. Active InBasket Time	39.57
6 Surgical	1/31/21	b. Active InBasket Time	43.67
6 Surgical	2/28/21	b. Active InBasket Time	45.49
6 Surgical	3/28/21	b. Active InBasket Time	44.01
6 Surgical	4/25/21	b. Active InBasket Time	46.34

6 Surgical	5/30/21	b. Active InBasket Time	42.38
6 Surgical	6/27/21	b. Active InBasket Time	42.82
6 Surgical	8/1/21	b. Active InBasket Time	45.08
6 Surgical	8/29/21	b. Active InBasket Time	44.39
6 Surgical	9/26/21	b. Active InBasket Time	45.74
6 Surgical	10/31/21	b. Active InBasket Time	43.24
6 Surgical	11/28/21	b. Active InBasket Time	43.64
6 Surgical	12/26/21	b. Active InBasket Time	42.33
6 Surgical	1/30/22	b. Active InBasket Time	45.27
6 Surgical	2/27/22	b. Active InBasket Time	43.61
6 Surgical	3/27/22	b. Active InBasket Time	44.56
1 Medical	7/28/19	c. EHR Work Outside of Work	193.56
1 Medical	9/1/19	c. EHR Work Outside of Work	194.05
1 Medical	9/29/19	c. EHR Work Outside of Work	193.53
1 Medical	10/27/19	c. EHR Work Outside of Work	193.58
1 Medical	12/1/19	c. EHR Work Outside of Work	183.42
1 Medical	12/29/19	c. EHR Work Outside of Work	193.76
1 Medical	1/26/20	c. EHR Work Outside of Work	203.36
1 Medical	3/1/20	c. EHR Work Outside of Work	202.02
1 Medical	3/29/20	c. EHR Work Outside of Work	190.14
1 Medical	4/26/20	c. EHR Work Outside of Work	194.52
1 Medical	5/31/20	c. EHR Work Outside of Work	208.31
1 Medical	6/28/20	c. EHR Work Outside of Work	206.22
1 Medical	7/26/20	c. EHR Work Outside of Work	205.79
1 Medical	8/30/20	c. EHR Work Outside of Work	208.97
1 Medical	9/27/20	c. EHR Work Outside of Work	210.10
1 Medical	11/1/20	c. EHR Work Outside of Work	203.48
1 Medical	11/29/20	c. EHR Work Outside of Work	206.84
1 Medical	12/27/20	c. EHR Work Outside of Work	213.69
1 Medical	1/31/21	c. EHR Work Outside of Work	224.57
1 Medical	2/28/21	c. EHR Work Outside of Work	227.45
1 Medical	3/28/21	c. EHR Work Outside of Work	214.70
1 Medical	4/25/21	c. EHR Work Outside of Work	220.35
1 Medical	5/30/21	c. EHR Work Outside of Work	212.66
1 Medical	6/27/21	c. EHR Work Outside of Work	212.96
1 Medical	8/1/21	c. EHR Work Outside of Work	216.31
1 Medical	8/29/21	c. EHR Work Outside of Work	214.95
1 Medical	9/26/21	c. EHR Work Outside of Work	221.60
1 Medical	10/31/21	c. EHR Work Outside of Work	204.00
1 Medical	11/28/21	c. EHR Work Outside of Work	213.53
1 Medical	12/26/21	c. EHR Work Outside of Work	209.52
1 Medical	1/30/22	c. EHR Work Outside of Work	222.32
1 Medical	2/27/22	c. EHR Work Outside of Work	222.17
1 Medical	3/27/22	c. EHR Work Outside of Work	219.37
2 Pediatric	7/28/19	c. EHR Work Outside of Work	106.48
2 Pediatric	9/1/19	c. EHR Work Outside of Work	96.33

2 Pediatric	9/29/19	c. EHR Work Outside of Work	101.62
2 Pediatric	10/27/19	c. EHR Work Outside of Work	93.94
2 Pediatric	12/1/19	c. EHR Work Outside of Work	93.39
2 Pediatric	12/29/19	c. EHR Work Outside of Work	97.71
2 Pediatric	1/26/20	c. EHR Work Outside of Work	105.92
2 Pediatric	3/1/20	c. EHR Work Outside of Work	94.21
2 Pediatric	3/29/20	c. EHR Work Outside of Work	85.92
2 Pediatric	4/26/20	c. EHR Work Outside of Work	99.74
2 Pediatric	5/31/20	c. EHR Work Outside of Work	112.72
2 Pediatric	6/28/20	c. EHR Work Outside of Work	104.52
2 Pediatric	7/26/20	c. EHR Work Outside of Work	109.54
2 Pediatric	8/30/20	c. EHR Work Outside of Work	103.37
2 Pediatric	9/27/20	c. EHR Work Outside of Work	105.89
2 Pediatric	11/1/20	c. EHR Work Outside of Work	106.18
2 Pediatric	11/29/20	c. EHR Work Outside of Work	106.17
2 Pediatric	12/27/20	c. EHR Work Outside of Work	110.53
2 Pediatric	1/31/21	c. EHR Work Outside of Work	115.11
2 Pediatric	2/28/21	c. EHR Work Outside of Work	127.39
2 Pediatric	3/28/21	c. EHR Work Outside of Work	115.96
2 Pediatric	4/25/21	c. EHR Work Outside of Work	119.51
2 Pediatric	5/30/21	c. EHR Work Outside of Work	113.23
2 Pediatric	6/27/21	c. EHR Work Outside of Work	117.70
2 Pediatric	8/1/21	c. EHR Work Outside of Work	125.92
2 Pediatric	8/29/21	c. EHR Work Outside of Work	113.39
2 Pediatric	9/26/21	c. EHR Work Outside of Work	119.96
2 Pediatric	10/31/21	c. EHR Work Outside of Work	111.45
2 Pediatric	11/28/21	c. EHR Work Outside of Work	114.90
2 Pediatric	12/26/21	c. EHR Work Outside of Work	110.97
2 Pediatric	1/30/22	c. EHR Work Outside of Work	128.92
2 Pediatric	2/27/22	c. EHR Work Outside of Work	129.57
2 Pediatric	3/27/22	c. EHR Work Outside of Work	123.82
3 Radiation	7/28/19	c. EHR Work Outside of Work	89.50
3 Radiation	9/1/19	c. EHR Work Outside of Work	86.96
3 Radiation	9/29/19	c. EHR Work Outside of Work	89.90
3 Radiation	10/27/19	c. EHR Work Outside of Work	86.90
3 Radiation	12/1/19	c. EHR Work Outside of Work	83.98
3 Radiation	12/29/19	c. EHR Work Outside of Work	86.11
3 Radiation	1/26/20	c. EHR Work Outside of Work	91.52
3 Radiation	3/1/20	c. EHR Work Outside of Work	97.88
3 Radiation	3/29/20	c. EHR Work Outside of Work	94.99
3 Radiation	4/26/20	c. EHR Work Outside of Work	92.68
3 Radiation	5/31/20	c. EHR Work Outside of Work	93.34
3 Radiation	6/28/20	c. EHR Work Outside of Work	90.34
3 Radiation	7/26/20	c. EHR Work Outside of Work	90.66
3 Radiation	8/30/20	c. EHR Work Outside of Work	92.41
3 Radiation	9/27/20	c. EHR Work Outside of Work	94.01

3 Radiation	11/1/20	c. EHR Work Outside of Work		89.84
3 Radiation	11/29/20	c. EHR Work Outside of Work		92.03
3 Radiation	12/27/20	c. EHR Work Outside of Work		95.41
3 Radiation	1/31/21	c. EHR Work Outside of Work		99.64
3 Radiation	2/28/21	c. EHR Work Outside of Work		100.43
3 Radiation	3/28/21	c. EHR Work Outside of Work		96.92
3 Radiation	4/25/21	c. EHR Work Outside of Work		96.48
3 Radiation	5/30/21	c. EHR Work Outside of Work		92.35
3 Radiation	6/27/21	c. EHR Work Outside of Work		93.44
3 Radiation	8/1/21	c. EHR Work Outside of Work		94.56
3 Radiation	8/29/21	c. EHR Work Outside of Work		92.35
3 Radiation	9/26/21	c. EHR Work Outside of Work		100.51
3 Radiation	10/31/21	c. EHR Work Outside of Work		93.59
3 Radiation	11/28/21	c. EHR Work Outside of Work		95.83
3 Radiation	12/26/21	c. EHR Work Outside of Work		94.69
3 Radiation	1/30/22	c. EHR Work Outside of Work		98.67
3 Radiation	2/27/22	c. EHR Work Outside of Work		98.15
3 Radiation	3/27/22	c. EHR Work Outside of Work		96.51
4 Gynecology	7/28/19	c. EHR Work Outside of Work		145.13
4 Gynecology	9/1/19	c. EHR Work Outside of Work		143.96
4 Gynecology	9/29/19	c. EHR Work Outside of Work		150.47
4 Gynecology	10/27/19	c. EHR Work Outside of Work		147.55
4 Gynecology	12/1/19	c. EHR Work Outside of Work		138.73
4 Gynecology	12/29/19	c. EHR Work Outside of Work		142.04
4 Gynecology	1/26/20	c. EHR Work Outside of Work		155.58
4 Gynecology	3/1/20	c. EHR Work Outside of Work		163.73
4 Gynecology	3/29/20	c. EHR Work Outside of Work		132.25
4 Gynecology	4/26/20	c. EHR Work Outside of Work		138.87
4 Gynecology	5/31/20	c. EHR Work Outside of Work		151.04
4 Gynecology	6/28/20	c. EHR Work Outside of Work		148.35
4 Gynecology	7/26/20	c. EHR Work Outside of Work		151.42
4 Gynecology	8/30/20	c. EHR Work Outside of Work		152.00
4 Gynecology	9/27/20	c. EHR Work Outside of Work		160.32
4 Gynecology	11/1/20	c. EHR Work Outside of Work		148.67
4 Gynecology	11/29/20	c. EHR Work Outside of Work		151.05
4 Gynecology	12/27/20	c. EHR Work Outside of Work		154.10
4 Gynecology	1/31/21	c. EHR Work Outside of Work		160.42
4 Gynecology	2/28/21	c. EHR Work Outside of Work		168.92
4 Gynecology	3/28/21	c. EHR Work Outside of Work		154.27
4 Gynecology	4/25/21	c. EHR Work Outside of Work		164.63
4 Gynecology	5/30/21	c. EHR Work Outside of Work		154.43
4 Gynecology	6/27/21	c. EHR Work Outside of Work		155.99
4 Gynecology	8/1/21	c. EHR Work Outside of Work		155.70
4 Gynecology	8/29/21	c. EHR Work Outside of Work		150.57
4 Gynecology	9/26/21	c. EHR Work Outside of Work		163.45
4 Gynecology	10/31/21	c. EHR Work Outside of Work		148.46

4 Gynecology	11/28/21	c. EHR Work Outside of Work	152.61
4 Gynecology	12/26/21	c. EHR Work Outside of Work	151.67
4 Gynecology	1/30/22	c. EHR Work Outside of Work	161.72
4 Gynecology	2/27/22	c. EHR Work Outside of Work	160.46
4 Gynecology	3/27/22	c. EHR Work Outside of Work	166.94
6 Surgical	7/28/19	c. EHR Work Outside of Work	137.17
6 Surgical	9/1/19	c. EHR Work Outside of Work	132.07
6 Surgical	9/29/19	c. EHR Work Outside of Work	136.27
6 Surgical	10/27/19	c. EHR Work Outside of Work	130.31
6 Surgical	12/1/19	c. EHR Work Outside of Work	122.57
6 Surgical	12/29/19	c. EHR Work Outside of Work	131.21
6 Surgical	1/26/20	c. EHR Work Outside of Work	139.14
6 Surgical	3/1/20	c. EHR Work Outside of Work	139.06
6 Surgical	3/29/20	c. EHR Work Outside of Work	95.14
6 Surgical	4/26/20	c. EHR Work Outside of Work	108.11
6 Surgical	5/31/20	c. EHR Work Outside of Work	131.02
6 Surgical	6/28/20	c. EHR Work Outside of Work	133.57
6 Surgical	7/26/20	c. EHR Work Outside of Work	139.92
6 Surgical	8/30/20	c. EHR Work Outside of Work	137.40
6 Surgical	9/27/20	c. EHR Work Outside of Work	138.00
6 Surgical	11/1/20	c. EHR Work Outside of Work	130.96
6 Surgical	11/29/20	c. EHR Work Outside of Work	130.40
6 Surgical	12/27/20	c. EHR Work Outside of Work	131.04
6 Surgical	1/31/21	c. EHR Work Outside of Work	145.48
6 Surgical	2/28/21	c. EHR Work Outside of Work	146.74
6 Surgical	3/28/21	c. EHR Work Outside of Work	141.48
6 Surgical	4/25/21	c. EHR Work Outside of Work	148.11
6 Surgical	5/30/21	c. EHR Work Outside of Work	139.05
6 Surgical	6/27/21	c. EHR Work Outside of Work	138.08
6 Surgical	8/1/21	c. EHR Work Outside of Work	144.76
6 Surgical	8/29/21	c. EHR Work Outside of Work	142.41
6 Surgical	9/26/21	c. EHR Work Outside of Work	145.17
6 Surgical	10/31/21	c. EHR Work Outside of Work	132.95
6 Surgical	11/28/21	c. EHR Work Outside of Work	135.14
6 Surgical	12/26/21	c. EHR Work Outside of Work	133.93
6 Surgical	1/30/22	c. EHR Work Outside of Work	145.26
6 Surgical	2/27/22	c. EHR Work Outside of Work	139.30
6 Surgical	3/27/22	c. EHR Work Outside of Work	147.23

Supplementary Table 3. Factors Associated with Oncologist Inbox and EHR Burden, Visit-Normalized Outcome Measures

	**Total Messages** (Messages per Visit)	**Patient Messages** (Messages per Visit)	**Total EHR Time** (Minutes per Visit)	**InBasket Time** (Minutes per Visit)	**EHR Time Outside Scheduled Hours** (Minutes per Visit)	**EHR Time on Unscheduled Days** (Minutes per Visit)	**EHR Work Outside of Work** (Minutes per Visit)
	Coefficient [95% CI]	Coefficient [95% CI]	Coefficient [95% CI]	Coefficient [95% CI]	Coefficient [95% CI]	Coefficient [95% CI]	Coefficient [95% CI]
**Sub-Specialty**							
Radiation Oncology	Ref.	Ref.	Ref.	Ref.	Ref.	Ref.	Ref.
Medical Oncology / Hematology	5.3*** [5.1; 5.4]	0.5*** [0.5; 0.5]	10.4*** [9.7; 11.2]	1.7*** [1.6; 1.8]	1.2*** [1.0; 1.3]	3.0*** [2.9; 3.2]	4.2*** [3.9; 4.5]
Pediatric Oncology	6.1*** [5.6; 6.6]	0.4*** [0.3; 0.5]	20.0*** [18.4; 21.7]	2.8*** [2.5; 3.1]	1.1*** [1.6; 2.3]	3.5*** [3.1; 3.9]	5.4*** [4.7; 6.1]
Gynecologic Oncology	4.4*** [4.1; 4.7]	0.4*** [0.3; 0.4]	4.4*** [3.2; 5.5]	1.7*** [1.5; 1.9]	0.1 [-0.2; 0.3]	3.3*** [2.1; 3.6]	3.4*** [2.9; 3.9]
Surgical Oncology	4.7*** [4.4; 5.1]	0.4*** [0.3; 0.4]	3.4*** [2.3; 4.6]	1.7*** [1.4; 1.1]	0.2 [-0.1; 0.4]	3.6*** [3.2; 3.1]	3.8*** [3.2; 4.3]
**Academic Status**							
Non-Academic Organization	Ref.	Ref.	Ref.	Ref.	Ref.	Ref.	Ref.
Academic Organization	1.0*** [0.8; 1.2]	-0.0 [-0.0; 0.0]	3.1*** [2.5; 3.8]	0.2** [0.1; 0.4]	-0.2** [-0.4; -0.1]	2.3*** [2.2; 2.5]	2.1*** [1.8; 2.4]
**Year**							
2019	Ref.	Ref.	Ref.	Ref.	Ref.	Ref.	Ref.
2020	-0.0 [-0.1; 0.1]	0.1*** [0.1; 0.1]	2.2*** [1.9; 2.5]	0.4*** [0.4; 0.5]	0.3*** [0.3; 0.4]	0.5*** [0.4; 0.6]	0.8*** [0.7; 0.9]
2021	0.1 [-0.0; 0.3]	0.1*** [0.1; 0.1]	1.6*** [1.2; 2.0]	0.6*** [0.5; 0.6]	0.3*** [0.2; 0.3]	0.6*** [0.5; 0.7]	0.9*** [0.7; 1.0]
2022	0.1 [-0.1; 0.3]	0.1*** [0.1; 0.1]	1.2*** [0.6; 1.7]	0.6*** [0.5; 0.7]	0.1* [0.0; 0.2]	0.6*** [0.5; 0.8]	0.7*** [0.5; 0.9]

Notes: N=380,878 observations for all models. * p <0.05 ** p <0.01 *** p < 0.001

Coefficients can be interpreted as the difference in either messages per visit or minutes per visit of EHR time compared to the reference category (e.g., radiation oncology). All dependent variables are normalized by visit volume, therefore models do not include a covariate for total visits. All models included calendar month fixed effects not shown to control for seasonality. Models include robust standard errors clustered at the physician level.

Supplementary Table 4. Factors Associated with Oncologist Inbox and EHR Burden – EHR Work Outside of Work Decomposition

	**EHR Work Outside of Work** (Minutes per Week)	**EHR Time Outside Scheduled Hours** (Minutes per Week)	**EHR Time on Unscheduled Days** (Minutes per Week)
	Coefficient [95% CI]	Coefficient [95% CI]	Coefficient [95% CI]
**Sub-Specialty**			
Radiation Oncology	Ref.	Ref.	Ref.
Medical Oncology / Hematology	107.7*** [102.3; 113.1]	42.8*** [39.5; 46.1]	62.5*** [59.7; 65.3]
Pediatric Oncology	44.9*** [36.7; 53.1]	17.8*** [12.9; 22.7]	23.9*** [19.7; 28.2]
Gynecologic Oncology	69.2*** [60.5; 77.9]	3.1 [-1.2; 7.4]	61.8*** [56.2; 67.5]
Surgical Oncology	55.5*** [47.4; 63.6]	1.6 [-2.6; 5.9]	49.9*** [44.9; 54.9]
**Academic Status**			
Non-Academic Organization	Ref.	Ref.	Ref.
Academic Organization	4.8 [-1.9; 11.6]	-21.1*** [-25.4; -16.9]	23.8*** [20.4; 27.2]
**Number of Visits**			
Total Weekly Visits (marginal effect of each additional visit)	1.7*** [1.4; 1.9]	1.5*** [1.3; 1.6]	0.3*** [0.2; 0.3]
**Year**			
2019	Ref.	Ref.	Ref.
2020	12.1*** [10.6; 13.6]	5.7*** [4.8; 6.6]	5.7*** [4.8; 6.6]
2021	19.2*** [17.0; 21.3]	8.0*** [6.7; 9.3]	10.6*** [9.2; 11.9]
2022	20.4*** [17.2; 23.6]	6.4*** [4.5; 8.3]	13.3*** [11.4; 15.2]

Notes: N=380,878 observations for all models. * p <0.05 ** p <0.01 *** p < 0.001

Coefficients can be interpreted as the difference in either weekly messages or minutes per week of EHR time compared to the reference category (e.g., radiation oncology). All dependent variables are measured weekly. All models included calendar month fixed effects not shown to control for seasonality. Models include robust standard errors clustered at the physician level.
